# Alternative Epigenetic Chromatin States of Polycomb Target Genes

**DOI:** 10.1371/journal.pgen.1000805

**Published:** 2010-01-08

**Authors:** Yuri B. Schwartz, Tatyana G. Kahn, Per Stenberg, Katsuhito Ohno, Richard Bourgon, Vincenzo Pirrotta

**Affiliations:** 1 Department of Molecular Biology and Biochemistry, Rutgers University, Nelson Laboratories, Piscataway, New Jersey, United States of America; 2 Department of Molecular Biology, Umeå University, Umeå, Sweden; 3 Computational Life Science Cluster, Umeå University, Umeå, Sweden; 4 European Bioinformatics Institute, Wellcome Trust Genome Campus, Hinxton, Cambridge, United Kingdom; Max-Planck-Institute of Immunobiology, Germany

## Abstract

Polycomb (PcG) regulation has been thought to produce stable long-term gene silencing. Genomic analyses in *Drosophila* and mammals, however, have shown that it targets many genes, which can switch state during development. Genetic evidence indicates that critical for the active state of PcG target genes are the histone methyltransferases Trithorax (TRX) and ASH1. Here we analyze the repertoire of alternative states in which PcG target genes are found in different *Drosophila* cell lines and the role of PcG proteins TRX and ASH1 in controlling these states. Using extensive genome-wide chromatin immunoprecipitation analysis, RNAi knockdowns, and quantitative RT–PCR, we show that, in addition to the known repressed state, PcG targets can reside in a transcriptionally active state characterized by formation of an extended domain enriched in ASH1, the N-terminal, but not C-terminal moiety of TRX and H3K27ac. ASH1/TRX N-ter domains and transcription are not incompatible with repressive marks, sometimes resulting in a “balanced” state modulated by both repressors and activators. Often however, loss of PcG repression results instead in a “void” state, lacking transcription, H3K27ac, or binding of TRX or ASH1. We conclude that PcG repression is dynamic, not static, and that the propensity of a target gene to switch states depends on relative levels of PcG, TRX, and activators. N-ter TRX plays a remarkable role that antagonizes PcG repression and preempts H3K27 methylation by acetylation. This role is distinct from that usually attributed to TRX/MLL proteins at the promoter. These results have important implications for Polycomb gene regulation, the “bivalent” chromatin state of embryonic stem cells, and gene expression in development.

## Author Summary

Polycomb (PcG) regulation has been thought to produce stable long-term gene silencing. Genomic analyses in *Drosophila* and mammals, however, have shown that it targets many genes that can switch state during development. Here we analyze the repertoire of alternative states in which PcG target genes are found in different *Drosophila* cell lines. In addition to the known repressed state, PcG targets can be found in a transcriptionally active state characterized by formation of an extended chromatin domain enriched in features that antagonize PcG repression. These features include the ASH1 protein, the N-terminal, but not C-terminal moiety of TRX, and histone H3K27 acetylation. ASH1/TRX N-ter domains and transcription are not incompatible with repressive marks, sometimes resulting in a “balanced” state modulated by both repressors and activators. Often, however, loss of PcG repression results instead in a “void” state, lacking transcription, H3K27acetylation, and other marks of active chromatin or binding of TRX or ASH1. The propensity of a target gene to switch states depends on relative levels of PcG, TRX, and activators.

## Introduction

The paradigmatic view of PcG repression is derived from the analysis of its role in the regulation of *Drosophila* homeotic (HOX) genes for which PcG genes were first discovered (for review see [1]). The expression pattern of HOX genes is set in the very early embryo by segmentation gene products, which determine the embryonic domains in which each HOX gene is active or repressed. The PcG proteins present in the early embryo do not prevent this initial activation but, when, shortly thereafter, the segmentation gene products disappear, they maintain the repressed state throughout development. While the analysis of HOX gene regulation gave the impression that PcG repression is all-or-nothing and, once established, is permanently maintained it is clear now that many other genes are also PcG targets in flies as in mammals, that PcG repression can set in at later stages and can be abrogated in the course of differentiation or in specific situations. The genetic evidence, however, shows that both the repressed and the non-repressed state tend to be inherited through successive cell cycles. The functions closely associated with the maintenance of the non-repressed state are those of the *trithorax* and *ash1* genes. These functions are not responsible for transcriptional activation per se, which still requires the appropriate enhancers and their binding factors, rather they are important to antagonize PcG repression and therefore to maintain a non-repressed “open” chromatin state that renders the target gene available for activation [2],[3].

How do TRX and ASH1 create a state resistant to PcG repression? Both TRX and ASH1 are SET domain proteins reported to have histone methyltransferase (HMTase) activity. Complexes containing TRX have been found to methylate histone H3K4 [4]. In budding yeast, Set1, a close relative of TRX, is a component of the COMPASS complex, which is recruited to the 5′ region of transcription units and methylates H3K4 to promote transcriptional elongation [5],[6]. Similar complexes containing mammalian TRX orthologues MLL1 and MLL2 have been biochemically characterized [7],[8]. Mammalian genomes as well as Drosophila encode several H3K4 methylating enzymes. Complexes containing true mammalian Set1 orthologues Set1A/B are responsible for most of the H3K4 methylation [9],[10]. It is thus likely that TRX and MLL1/2 complexes are specialized versions recruited to a subset of genes that includes PcG target genes. TRX and its mammalian counterparts are cleaved in two moieties by the threonine aspartase Taspase1, of which the C-terminal moiety contains the catalytic SET domain [11]–[13]. In the case of MLL1, the two cleavage products have been found associated together [14]. ASH1 has been variously reported to methylate *in vitro* H3K4, H3K9, H4K20 [15] or H3K36 [16].

The hallmark of a locus repressed by PcG mechanisms is its residence within a broad chromatin domain enriched in trimethylated H3K27, often including more than one gene [17]–[21]. PcG repression and H3K27 trimethylation are initiated and maintained by one or more Polycomb Response Elements (PREs) that appear as narrow regions, within the methylation domain, that simultaneously bind several PcG proteins [17]–[19]. In mammalian embryonic stem cells, many PcG target genes have been reported to bear both repression-associated marks and H3K4me3 [22]. These genes have been said to assume a bivalent state with some transcriptional activity taking place even in the presence of PcG complexes. Upon differentiation, these bivalent states may become resolved into fully active states, with no PcG proteins, or fully repressed states, with PcG binding and extensive H3K27me3 domains. What controls the balance between activity and repression and whether bivalent states occur in *Drosophila* is not known. Certain *Drosophila* genes have been found to bind PcG proteins while remaining functional and sustaining transcriptional activity [19]. These findings raise questions about the relationship between PcG complexes and associated chromatin marks and transcription, Trithorax Group (TrxG) proteins and their associated chromatin marks. In this work, we have exploited the genomic approach to seek answers to these questions. By comparing PcG/TrxG and transcriptional landscapes in three Drosophila cultured cell lines of different origin we have asked what range of chromatin states can be assumed by PcG target genes, whether the binding of PcG proteins constitutes the default state and how the chromatin landscape and the binding of TRX and ASH1 is involved in changes and in the stable maintenance of alternative states.

## Results

To screen for alternative chromatin states of PcG target genes we determined the genomic distributions of PC, E(Z) and H3K27me3 in three different cultured cell lines: ML-DmBG3-c2 cells (hereafter BG3 cells) derived from larval brain and ventral ganglion, ML-DmD23-c4 cells from imaginal wing disc (hereafter D23 cells) and Sg4 cells (previously reported in [19]), derived from the embryonic Schneider L2 line, using chromatin immunoprecipitation analysed by hybridization to Drosophila genomic tiling arrays (ChIP/chip).

We identified PcG target regions, as domains enriched in H3K27me3 and containing a site that binds both PC and E(Z) by the stringent criterion of two-fold enrichment. We found 99 such domains in Sg4 cells, 107 domains in BG3 cells and 89 domains in D23 cells. These we call Class I PcG target regions (Text S1, Table S1). They include classical examples of genes whose PcG regulation is known from genetic studies, the genes of the *Bithorax* and *Antennapedia Complexes*, *engrailed*, and *hedgehog*. In addition, each cell line had a category of regions (Class II PcG target regions, Table S1), that contained PC and H3K27me3 but little or no detectable E(Z). The binding of PC and H3K27me3 in these regions is weaker and the methylation domains are less broad (Figure S1). Here we will limit our analysis to Class I regions (hereafter PcG target regions) as these represent loci whose PcG regulation is confirmed by genetic evidence.

Pairwise comparison of PcG target regions detected in any two cell lines shows that their repertoire is relatively stable but we found a total of 53 cases in which PcG protein binding and H3K27me3 was lost in one of the lines (Figure S2). Each cell line contributes additional genes to the catalogue of PcG target genes (Figure S3, Tables S2, S3, S4, S5). Thus, the third cell line still contributes about 25% more genes to the number of potential PcG targets, suggesting that at least 25% more remain to be identified in the *Drosophila* genome.

### Repressed and active chromatin states of the *Abdominal-B* gene

We then asked whether the differences in PcG binding in the three cell lines were reflected by changes in transcriptional activity of the underlying genes and/or in the binding of TrxG proteins. In the analyses that follow, we used the presence of RNA Pol II at the Transcription Start Site (TSS) and H3K4me3 at position +500 bp as criteria to distinguish transcriptionally active from inactive genes (for details, see Text S1 and Figure S4).

**Figure 1:**
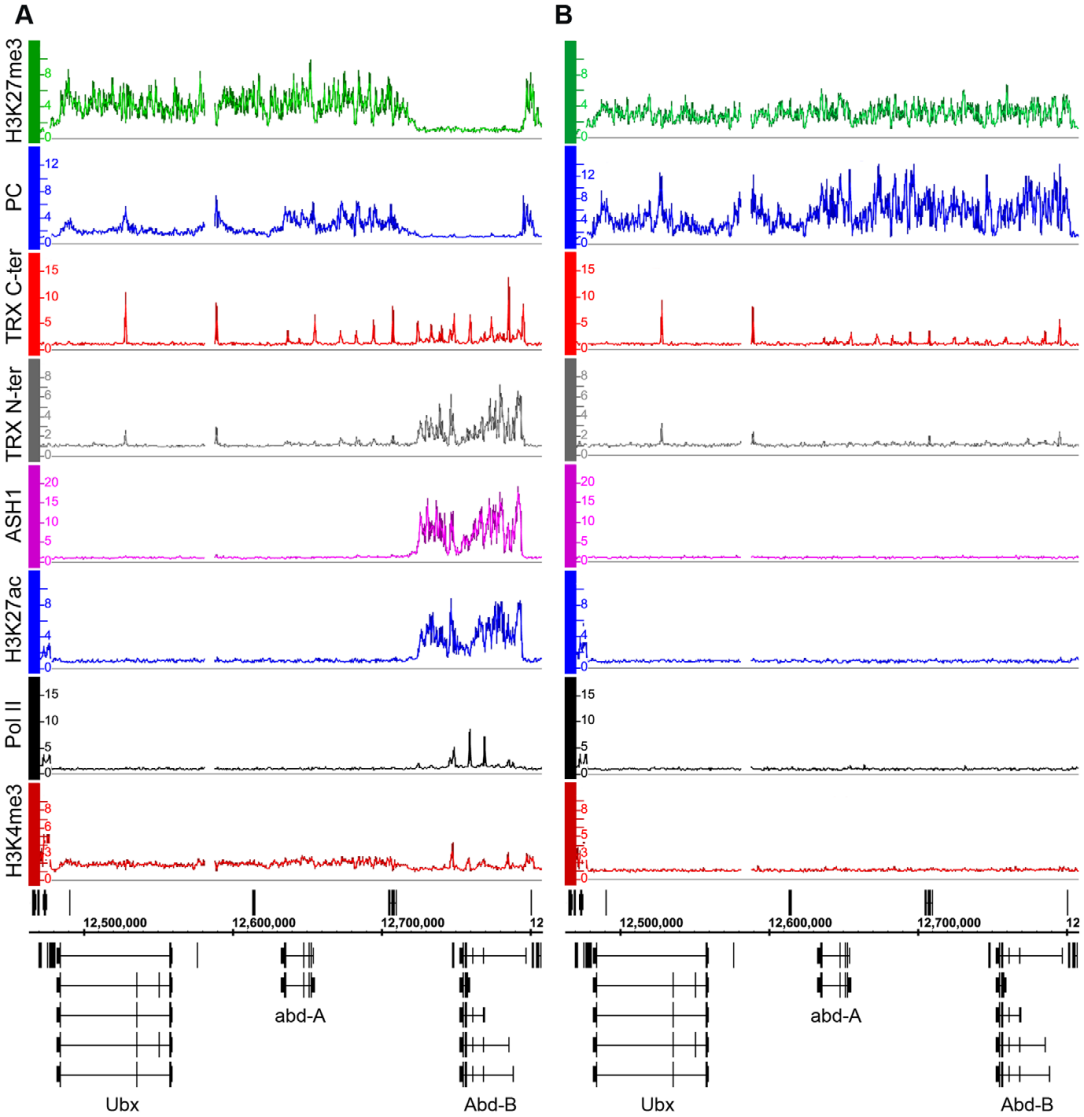
The *Abd-B* gene is repressed in BG3 cells and active in Sg4 cells. The distributions of PcG, TrxG, ASH1, Pol II, and associated histone marks in the Bithorax Complex were mapped in (A) Sg4 and (B) BG3 cells. ChIP–chip results with antibodies indicated on the left of the graphs were expressed as smoothed ChIP/Input signal ratios averaged for two independent experiments. The positions and the exon structure of annotated transcripts are shown above (transcription left to right) and below (transcription right to left) the coordinate scale (in bp).

We first consider the *Bithorax Complex* (*BX-C*), containing the three homeotic genes *Ubx*, *abd-A* and *Abd-B*. In two cell lines, BG3 and D23, all three genes are PcG-repressed: they bind PcG proteins at the known or presumed PREs and the entire BX-C domain of more than 320 kb is highly enriched for H3K27me3 (Figure 1B, Figure S5). In Sg4 cells, *Ubx* and *abd-A* are repressed but *Abd-B* is transcriptionally active, lacks H3K27me3 and at least two of its known PREs, *Fab-7* and *Fab-8*, lack PcG binding (Figure 1A). *Abd-B* has five different promoters. In Sg4 cells four are active, while the one furthest upstream is inactive, binds PcG proteins and is enriched for H3K27me3 [19]. Consistent with this, peaks of Pol II and of H3K4me3 are found at the active *Abd-B* promoters, as well as in the *Abd-B* downstream region, known to produce non-coding RNAs [23] (Figure 1A). These peaks are absent in BG3 and D23 cells where the repressive marks predominate (Figure 1B, Figure S5).

We next determined the relationship of TRX and ASH1 to the state of activity of the *BX-C* genes using two sets of antibodies for each of the N-ter TRX, the C-ter TRX and the ASH1 polypeptides (see Text S1 and Table S9 for details). When assayed with C-ter antibodies, TRX is found at the known and presumptive PREs of the *BX-C* region, forming sharp and distinctive peaks irrespective of the transcriptional activity of the target genes, consistent with previous observations [17],[18],[24],[25]. The TRX C-ter antibodies also detected TRX at transcriptionally active promoters in the *Abd-B* region of Sg4 cells but not in BG3 or D23 cells or at the repressed promoters of *Ubx* and *abd-A* (Figure 1, Figure S5).

The TRX N-ter distribution shows surprising differences. It is found together with TRX C-ter at PREs and at active promoters but it is also associated with the broad region that includes *Abd-B* and its downstream non-coding transcribed region in Sg4 but not in BG3 cells (Figure 1). A related function is implied by the distribution of ASH1. No ASH1 was found in the entire BX-C region of BG3 or D23 cells, where all the genes are PcG-repressed, but in Sg4 cells ASH1 was extensively associated with the entire region occupied by TRX N-ter (Figure 1).

These findings suggest that full derepression results in massive loss of H3K27me3, dissociation of Polycomb proteins from PREs and formation of an extensive ASH1 domain. They also point to a specific function of N-ter TRX that does not involve HMTase activity but extends with ASH1 over the entire domain that would otherwise be enriched for H3K27me3.

### ASH1 and TRX N-ter domains mark the active chromatin state of PcG targets

TRX was found at all PREs of the *Bithorax-Complex*. Of 170 computationally defined PREs in Sg4 cells (see Text S1 and Table S6 for details) 94% bind TRX C-ter, indicating that the presence of TRX at PREs is a common feature of PcG-repressed genes. The N-ter TRX antibody is generally weaker in ChIP-chip experiments and often does not reach the two-fold enrichment cut-off. Despite this, 39% of computational PREs also bind TRX N-ter, suggesting that the TRX complex at PREs contains both TRX moieties. Furthermore, looking at the entire catalogue of potential PREs, we found that TRX binds at essentially all known or presumptive PREs, whether or not they were also occupied by PcG proteins. That is, irrespective of the repressed or active state of the associated genes, PREs are also TREs (Trithorax Response Elements).

**Figure 2:**
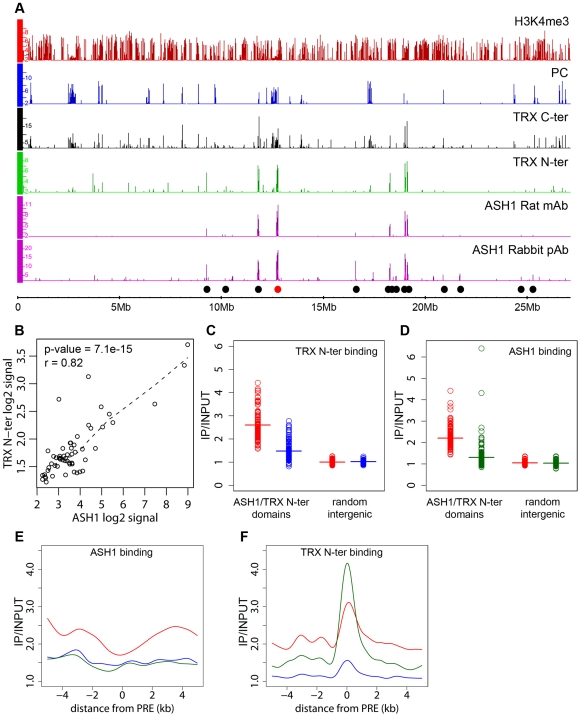
ASH1/TRX N-ter domains. (A) Distributions of indicated proteins on chromosome 3R of Sg4 cells were plotted at 2-fold enrichment cutoff. Independent antibodies detect ASH1 at a small number of sites all of which also bind TRX. Only sites detected by both anti-ASH1 antibodies (black dots) were used for further analysis. The red dot marks the position of the *Abd-B* locus. Nearly all strong TRX binding sites that do not bind ASH1 correspond to repressed PC targets. Note that ASH1 or TRX bind to a small subset of active genes marked by H3K4me3. (B) The average enrichment of TRX N-ter was plotted against that of ASH1 for each ASH1 binding region in Sg4 cells. The dashed line shows the lowess fitting of the data. The Pearson product moment correlation test shows that the extent of binding of TRX N-ter and ASH1 is highly correlated. Comparison of average enrichment of TRX N-ter (C) and ASH1 (D) within bound regions in untreated BG3 cells (red) and BG3 cells treated with dsRNA against TRX (blue) or ASH1 (green) shows significant reduction of protein binding after RNAi. The background enrichment, assayed in 100 randomly selected intergenic regions, remains unchanged. Bars indicate the sample means. RNAi affects the distributions of ASH1 (E) or TRX N-ter (F). The plots show cubic spline fitting of superposed data from 10 kb windows centered at 19 computational PREs, within ASH1/TRX N-ter domains in BG3 cells before (red) or after TRX (blue) or ASH1 (green) RNAi. ASH1 shows no preferential binding to PREs and knock-down of ASH1 or TRX results in the uniform reduction of ASH1 binding throughout the domains. In contrast ASH1 knock-down removes TRX N-ter from the domains but not from the PREs.

The presence of an ASH1/TRX N-ter domain at the derepressed *Abd-B* locus is consistent with the antagonistic genetic interactions between *PcG* and *ash1* or *trx*. Are the ASH1/TRX N-ter domains generally characteristic of PcG target loci in the derepressed state? A survey of the Sg4 genome revealed 56 ASH1 domains half of which were broad: ranging from 10.7 to 77.5 kb in length (Figure 2A). 79% of the ASH1 binding regions also bound TRX N-ter. An excellent overall correlation between the binding levels of the two proteins within ASH1/TRX N-ter domains (Figure 2B) suggested that their binding is interdependent. To test this directly we knocked down ASH1 or TRX in BG3 cells to ∼20% of wild type by RNAi (Figure S6B) and assayed the chromosomal distribution of ASH1 and TRX N-ter by ChIP/chip. The knock-downs of both protein resulted in substantial reduction of their binding to chromosomes (Figure 2C and 2D). The knock-down of TRX also reduced ASH1 binding to an extent comparable with that seen in RNAi against ASH1 itself (Figure 2E). Strikingly, RNAi against ASH1 led to marked loss of TRX N-ter from the broad ASH1/TRX N-ter domains but not from presumptive PREs (Figure 2F). We therefore conclude that binding of ASH1 and TRX N-ter within broad domains is interdependent while association of TRX with PREs is not dependent on ASH1.

**Figure 3:**
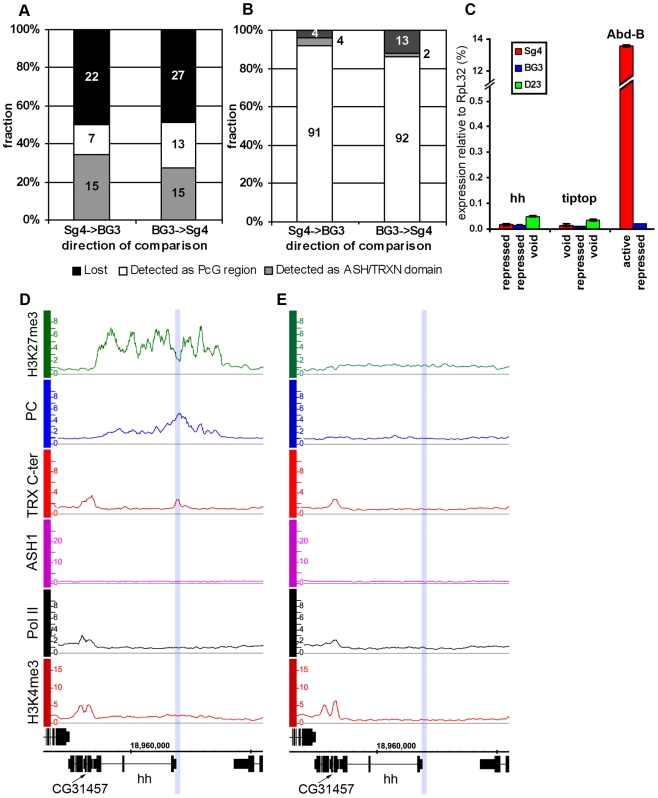
The “void” chromatin state. Changes of ASH1/TRX N-ter domains (A) and PcG target regions (B) between Sg4 and BG3 cells indicate the existence of the chromatin state devoid of both PcG and TrxG proteins. The y-axis shows the fraction of regions changed while their absolute numbers are indicated within bars. (C) The expression of *hh*, *tiptop*, and *Abd-B* loci in Sg4 (red), BG3 (blue), or D23 cells (green) was assayed by qRT–PCR. In this and following figures the histogram shows the mean of two independent experiments with error bars indicating the scatter. Comparison with *Abd-B*, which is active in Sg4 and repressed in BG3 cells, shows that in the “void” state *hh* and *tiptop* remain transcriptionally inactive. (D) The *hedgehog* (*hh*) gene is PcG-repressed in Sg4 cells, with PC and TRX bound at a previously identified PRE [26] (blue shade), but is in the “void” state in D23 cells (E), with no PC nor H3K27me3 but also lacking TRX, ASH1, Pol II, and H3K4me3.

We next examined the correlation between transcriptional activity of PcG target genes and binding of ASH1/TRX N-ter. When we looked at genes that are PcG-repressed in one cell line but acquire Pol II at TSS and H3K4me3 at position +500 in another cell line, in 39 of 47 cases (83%) derepression was accompanied by binding of ASH1, indicating strong correlation. We then asked if regions that bind ASH1/TRX N-ter in Sg4 cells but no longer do so in BG3 cells generally acquire PcG and H3K27me3 and vice versa. From 44 regions that bind ASH1 and TRX N-ter in Sg4 cells, 29 no longer bind these proteins in BG3 cells and seven of these simultaneously acquire PC and H3K27me3 (Figure 3A). Conversely when the state of PcG targets detected in Sg4 cells was examined in BG3 cells, half of the cases that lost PcG binding acquired at the same time an ASH1/TRX N-ter domain (Figure 3B). The reciprocal comparison between BG3 and Sg4 cells gave similar results. Although our current data set does not provide conclusive proof that all genomic ASH1/TRX N-ter domains correspond to PcG targets, we conclude that the chromatin state of the active *Abd-B* locus is not exceptional and that in multiple instances the loss of PcG and H3K27me3 in one of the cell lines was accompanied by the appearance of an ASH1 domain and derepression, in turn suggesting that broad binding of ASH1/TRX N-ter is likely a general mark of the derepressed state of PcG targets.

### PcG target genes in the “void” chromatin state

The comparison of PcG and trxG landscapes in different cell lines showed that in half of the cases the loss of PcG and H3K27me3 was not associated with the gain of an ASH1/TRX N-ter domain and vice versa (Table S7). Strikingly, in these cases TRX binding to the PRE was also absent. In 31 of 36 cases (86%), genes that lost PcG and H3K27me3 without acquiring ASH1/TRX N-ter did not display marks of transcriptional activity in the absence of PcG repressive marks.

This chromatin state is vividly exemplified by the *hedgehog* (*hh*) locus in D23 cells (Figure 3E). *hh* encodes an important signaling protein essential for morphogenesis in flies and mammals, whose control by PcG mechanisms is well established [26]. Consistent with this, *hh* is a PcG target region in Sg4 (Figure 3D) and BG3 (not shown) cells. However, in D23 cells this locus was completely devoid of both PcG and TrxG proteins but remained transcriptionally inactive (Figure 3C).

Another example of this kind is *tiptop* (*tio*), also an essential developmental gene. In this case PcG regulation of *tio* was evident in the BG3 line (Figure S7B) but in Sg4 (Figure S7A) and D23 cells (not shown) the gene is devoid of both PcG and TrxG proteins. As in the case of *hh*, the gene remained inactive in the absence of PcG proteins (Figure 3C). We call this chromatin state the “void” state since it is characterized by the absence of PcG/TrxG proteins or chromatin marks. The lack of transcriptional activity of target genes in the “void” state indicates that mere absence of PcG proteins is not sufficient for expression of the target gene. Conversely, lack of transcriptional activity or of active chromatin marks is not sufficient for binding of PcG proteins to a PcG target gene. It is clear that neither binding of PcG proteins nor of TRX constitutes the default chromatin state of PcG target genes.

### Anti-PcG function of ASH1-TRX domains

**Figure 4:**
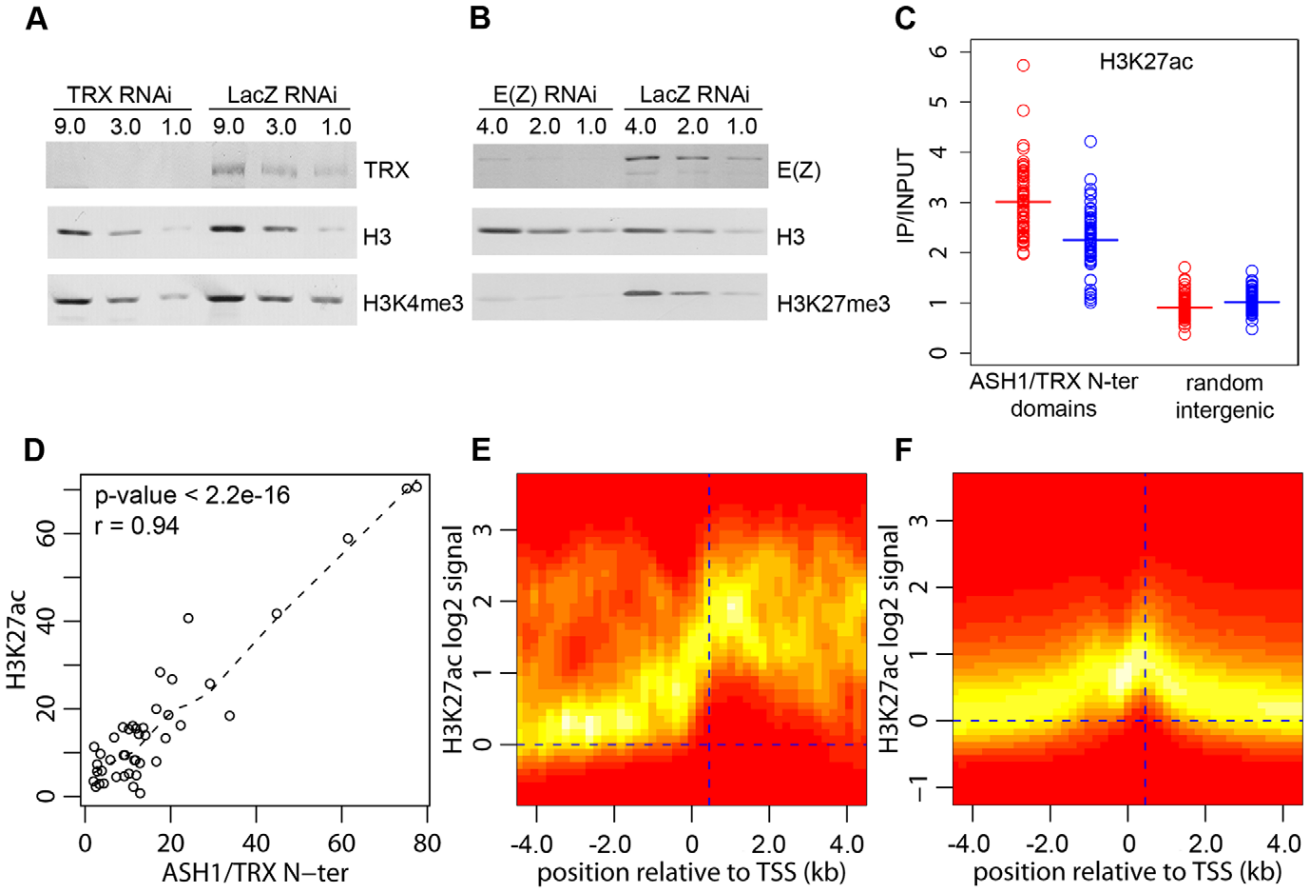
H3K27ac and ASH1/TRX N-ter domains. (A) Effect of TRX or control LacZ RNAi knockdown on H3K4me3 compared to (B) the effect of E(Z) knockdown on H3K27me3. The western blots were probed with antibodies indicated on the right. (C) Average enrichment of H3K27ac within ASH1/TRX N-ter domains (circles) after TRX RNAi (blue) or control LacZ RNAi (red) compared to that in 100 randomly selected intergenic regions (squares). Bars indicate the sample means. (D) Scatter plot of lengths (in kb) of corresponding H3K27ac and ASH1/TRX N-ter binding regions. Dashed line shows the lowess fitting of the data. The Pearson product moment correlation test shows that ASH1/TRX N-ter domains are coextensive with H3K27ac. The TSS of active transcription units of genes with (E) or without ASH1/TRX N-ter domains (F) were defined based on Pol II and H3K4me3 binding. The log_2_-transformed H3K27ac/Input ratios from 10 kb windows centered on these TSS were superimposed into a single scatter plot. The color (red = zero, white = highest) indicates the density of observations. The plots show that H3K27ac is extensively distributed in ASH1/TRX N-ter domains but is confined to the region around position +450 (vertical dashed line) downstream of the TSS of active genes lacking ASH1. The weak peak of H3K27ac upstream of TSS is due to the frequency of closely juxtaposed divergently transcribed genes.

The function of TRX and ASH1 is to antagonize PcG repressive activities when the target gene is in the active mode. Genetic evidence indicates that, in the absence of PcG repression, TRX and ASH1 are dispensable for the expression of PcG target genes, suggesting that their role is different from that of general transcription activators [3]. Consistent with this, our genome-wide mapping indicates that ASH1 and TRX are not general transcription factors. Thus, while Sg4 cells have 5771 active transcription units, they contain only 56 ASH1 domains and 618 TRX binding regions, implying that transcription of the vast majority of genes does not involve ASH1 or TRX. Our data also argue against the idea that TRX is a functional ortholog of the yeast SET1 protein, which is generally recruited to active promoters via its interaction with RNA Pol II and is responsible for trimethylation of H3K4 on promoter-proximal nucleosomes [6]. Most active promoters bearing the H3K4me3 mark have no TRX binding (Figure 2A) and we did not detect any decrease in the overall level of H3K4me3 after ten cell generations of RNAi treatment reducing the level of TRX to 20% of wild type (Figure 4A). A similar knockdown of E(Z) produced prominent reduction in global levels of H3K27me3 (Figure 4B).

H3K4 methylation by TRX does not explain its anti-repressive activity: H3K4 methylation by other methyltransferases occurs at all active loci. Furthermore, TRX N-ter lacks the SET domain and therefore has no methyltransferase activity. ASH1 does have a SET domain but our results show that ASH1-TRX binding regions are not domains of H3K4 methylation. A histone modification that would be clearly antagonistic to PcG mechanisms is H3K27 acetylation (H3K27ac). In fact all ASH1/TRX N-ter domains are co-extensive with domains of H3K27ac (Figure 4D, Figure 1A) which reach both upstream and downstream of target genes. This modification is not exclusive for ASH1/TRX N-ter domains and is also found at 66% of transcriptionally active genes that lack ASH1. In these cases, however, the H3K27ac distribution is narrow (compare Figure 4E and 4F) and largely confined to a prominent peak 450 bp downstream of the promoter. The histone acetyltransferase (HAT) activity responsible for the broad H3K27ac distribution in ASH1/TRX N-ter domains appears to be specific: H3K9ac in these domains is limited to a peak around 450 bp downstream of the promoter and is no different from that found at other active genes (Figure S8).

Coextensive binding of ASH1/TRX N-ter and H3K27ac suggests that HAT activity responsible for H3K27 acetylation may constitute a part of ASH1 or/and TRX N-ter protein complexes. To explore this possibility we examined the effect of TRX knock-down on the level of H3K27ac within ASH1/TRX N-ter domains. The knock-down of TRX results in simultaneous loss of ASH1 thus the effect of loss of both proteins was assayed in this experiment. Only a modest decrease in H3K27ac was found (Figure 4C), despite the strong reduction in TRX N-ter and ASH1 (Figure 2E). We conclude that the level of H3K27ac within ASH1/TRX N-ter domains is functionally related to the binding of TRX or ASH1 but is not directly linked to their levels.

### PcG target genes in balanced chromatin states

**Figure 5:**
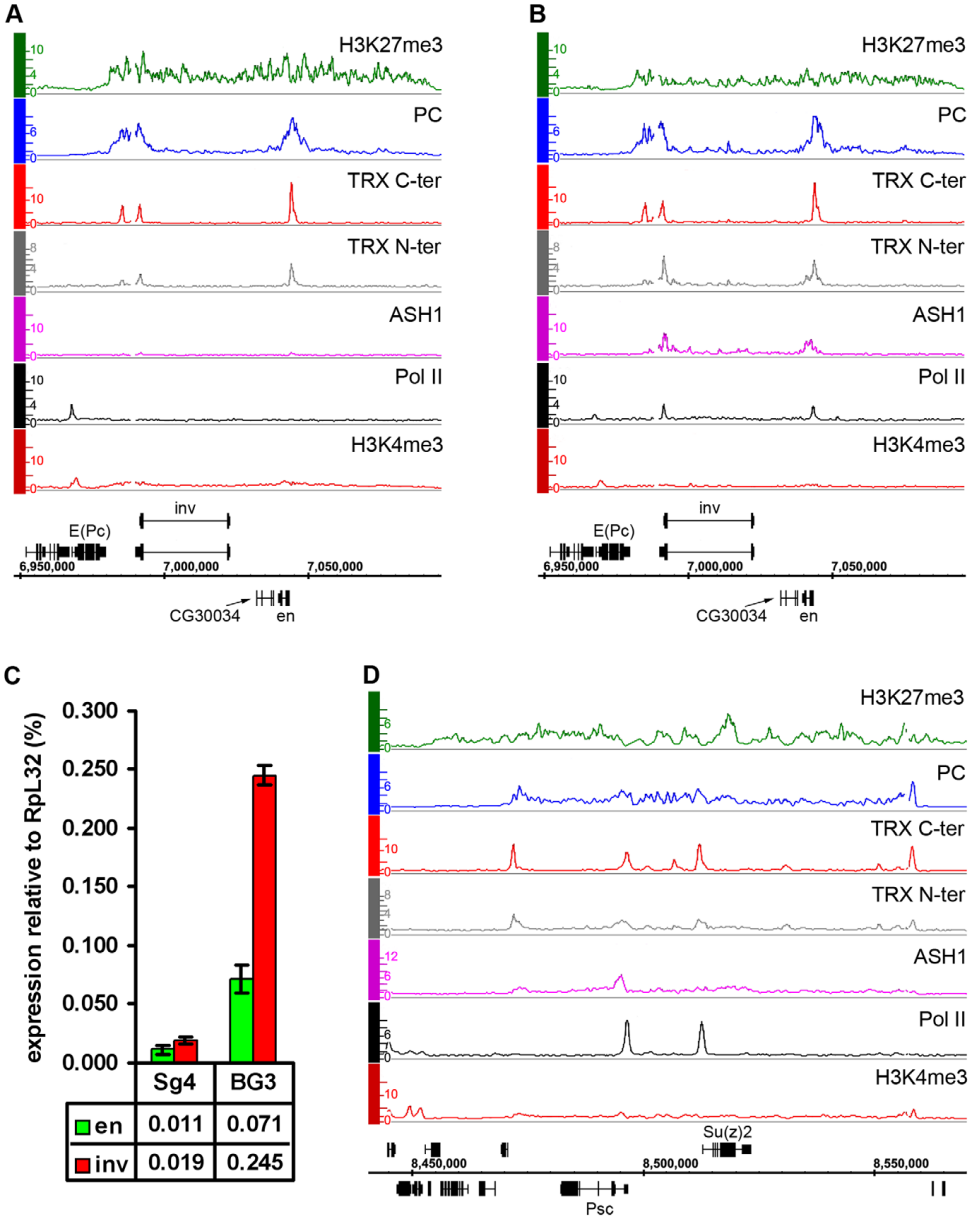
Examples of balanced chromatin state. (A) The *en-inv* locus in Sg4 cells is repressed and silent, with TRX binding at three known PREs. (B) in BG3 cells both genes still bind PC and are H3K27 methylated but at the same time bind Pol II and ASH1 and produce ∼10 times more transcripts. (C) Results of qRT–PCR analysis of *en* and *inv* expression in Sg4 and BG3 cells. (D) The *Psc-Su(z)2* locus shows profiles of H3K27me3 and PC over a broad domain yet it also binds RNA Pol II at the *Psc* and *Su(z)2* promoters and localized peaks of TRX and ASH1, consistent with expression of both genes.

Consistent with the idea that ASH1 is a marker of anti-repressive, TrxG-dominated chromatin states, there is little overlap between regions bound by ASH1 and domains characterized by H3K27me3 and PcG proteins (∼6%) and, unlike TRX, ASH1 is not generally recruited to PREs in the repressive state. However, exceptional sites that bind both ASH1 and H3K27me3 are very instructive (Table S8). One of these corresponds to the *Psc* gene, which encodes a core component of the PRC1 complex. In all cell lines examined, this gene has prominent PcG binding and H3K27me3 but remains functionally active and produces PSC protein. A prominent peak of Pol II is found at the TSS of *Psc* and of the adjacent, closely related and divergently transcribed *Su(z)2* gene. The presence of ASH1 and TRX N-ter in a narrow region at the 5′ end of *Psc* (Figure 5D) supports the idea that these proteins mark alleviation of transcriptional repression at PcG target genes. It also suggests that PcG and TrxG proteins at this locus are not mutually exclusive, although it remains possible that *Psc* expression cycles on and off.

Another region of this kind contains *engrailed* (*en*) and *invected* (*inv*), two genes encoding related homeodomain factors with important roles in metazoan development. Their regulation by PcG mechanisms is well known [27]. In Sg4 and D23 cells, both genes are contained within a PcG domain and lack Pol II at their promoters (Figure 5A, Figure S9). However, in BG3 cells, in addition to PcG and H3K27me3, both genes show pronounced binding of ASH1 and TRX N-ter around the 5′ ends of their transcription units as well as prominent peaks of Pol II at promoters (Figure 5B). In agreement with this, both genes produce elevated levels of mRNA in BG3 cells relative to Sg4 (Figure 5C).

The above examples indicate that, while most PcG/TrxG target loci in cultured cells are dominated by either the PcG or TrxG function, some genes display both simultaneously, or at least they can alternate from one state to the other. We call such a condition a “balanced” state and speculate that in this state PcG and TrxG proteins act in concert with other positive and negative regulators of transcription, keeping one epigenetic activity from “overcoming” the other.

### Derepression of PcG target genes is linked to the switching of chromatin state

To what extent are the alternative chromatin states described above dependent on the relative cellular levels of PcG and TrxG proteins? Do these levels directly determine their binding equilibrium at target genes? To address these questions we looked for changes in the genomic distributions of PC, TRX N-ter, ASH1, H3K27me3 and H3K27ac in BG3 cells subjected to RNAi against TRX or PC.

**Figure 6:**
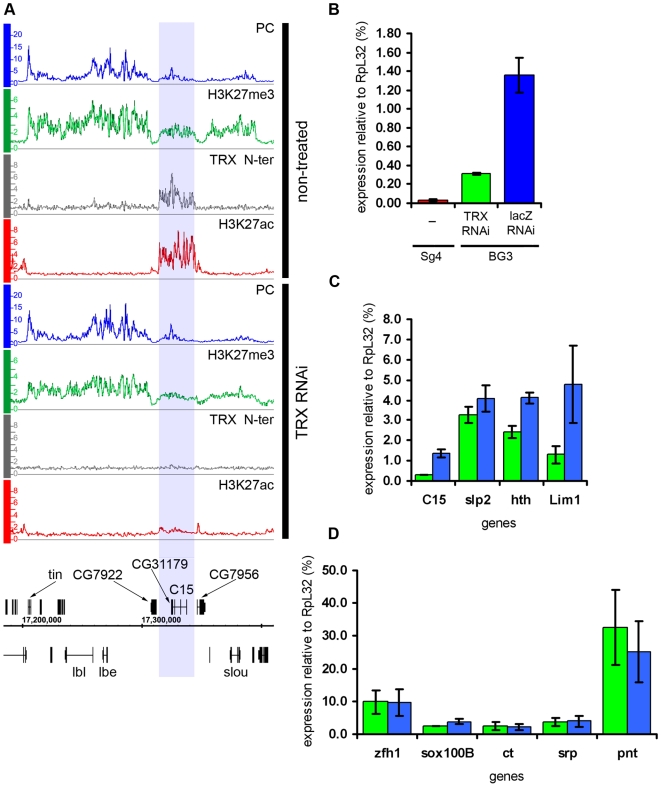
Effects of TRX knock-down. (A) An example of exceptionally strong depletion of H3K27ac (blue shade) at the *C15* locus after TRX RNAi. (B) qRT–PCR analysis indicates that the expression of *C15* is reduced after TRX RNAi in BG3 cells, however, it remains 10 times higher then in Sg4 cells where the gene is fully repressed by PcG [19]. (C) The expression of loci that show profound depletion of H3K27ac after TRX RNAi is slightly lower (green) than in mock-treated cells (blue). (D) TRX RNAi has no effect on the transcription of representative active PcG target genes.

The knock-down of TRX led to a substantial drop in the amount of TRX N-ter and ASH1 bound to transcriptionally active PcG targets (Figure 2C and 2D). This, however, caused no redistribution of either PC or H3K27me3, leading us to conclude that the overall relationship of PcG and ASH1/TRX N-ter binding to target genes is not governed by a simple competitive equilibrium. Consistent with the lack of changes in the distributions of PcG proteins, the expression of target genes in the “active” chromatin state was not altered (Figure 6D), indicating that once a stable chromatin state is established, at least over 9 generations in this cell culture, the expression of active PcG target genes is not very sensitive to the levels of associated ASH1 and TRX N-ter. While depletion of TRX had modest general effect on the levels of H3K27ac at active PcG targets, four regions showed exceptionally strong loss of K27 acetylation (Figure 6A). Curiously, all four resided in the “balanced” state in untreated BG3 cells but, despite the initial presence of PcG proteins and H3K27me3, the nearly complete loss of H3K27ac after TRX RNAi did not cause more binding of PcG or H3K27me3. Expression was modestly reduced at all four regions (Figure 6C) but not completely repressed (Figure 6B). We conclude that, while the level of H3K27ac in ASH1/TRX N-ter domains is positively correlated with the extent of transcriptional activity, it is not its primary determinant. These observations also suggest that H3K27ac is not the only anti-PcG mark.

**Figure 7:**
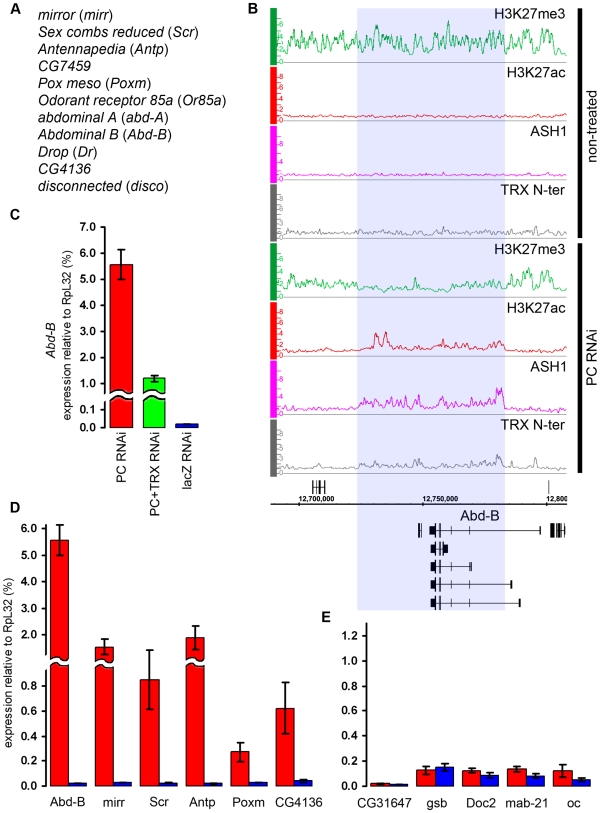
The switching of chromatin state induced by PC RNAi. (A) Genes that switch state from “repressed” to “balanced” after PC RNAi in BG3 cells. (B) The changes in the distribution of chromatin proteins and marks at the *Abd-B* locus after PC RNAi in BG3 cells. The affected region is marked by blue shade. (C) The expression of *Abd-B* after PC RNAi (red bar), double PC+TRX RNAi (green bar) or mock lacZ RNAi (blue bar) was assayed by qRT-PCR using primers that amplify the common part of the *Abd-B* transcripts. (D) Expression of representative genes from (A) assayed by qRT–PCR in cells subjected to PC RNAi (red bars) or mock lacZ RNAi (blue bars). (E) Expression of representative “repressed” genes under the same conditions as in (D).

Similar to TRX knock-down, the reduction of PC levels to 20% of wild type (Figure S6) did not change the chromatin states of the majority of the target genes. Despite the lack of global effect, we found eleven exceptional PcG-repressed loci which, upon PC knockdown, acquired binding of ASH1, TRX N-ter, H3K27ac and a corresponding marked reduction of H3K27me3, i.e. they switched their chromatin state from “fully repressed” to “balanced” (Figure 7A and 7B). When expression of six of these loci was checked by RT-qPCR, switching of the chromatin state was paralleled in all cases by strong increase in transcription (Figure 7D). In marked contrast, the expression of randomly chosen PcG target genes whose repressed chromatin state did not change remained constant (Figure 7E). We interpret these findings to indicate that in BG3 cells the transcriptional activators of the exceptional 11 PcG target genes are available but at levels below the threshold necessary for derepression under normal PC levels. PC knockdown lowers the threshold for the amount of activator required to switch to a transcriptionally active chromatin state. We also conclude that derepression of PcG target genes is tightly linked to the formation of an ASH1/TRX N-ter domain and appearance of the H3K27ac mark.

In flies, *trx* mutations act as suppressors of *Pc* mutations. We thus wondered whether the derepression of target genes caused by the reduction of PC levels in the cell culture is also sensitive to the level of TRX. Comparison of the effects of single PC knockdown versus simultaneous knockdown of PC and TRX on the expression levels of *Abd-B* and *mirr* genes indicates that this is indeed the case. Double knockdown of PC and TRX resulted in two- to four-fold lower transcription than knockdown of PC alone (Figure 7C, Figure S10), suggesting that, for a given dose of activator, the response of the target gene depends on the relative levels of PC and TRX.

## Discussion

### Alternative states

**Figure 8:**
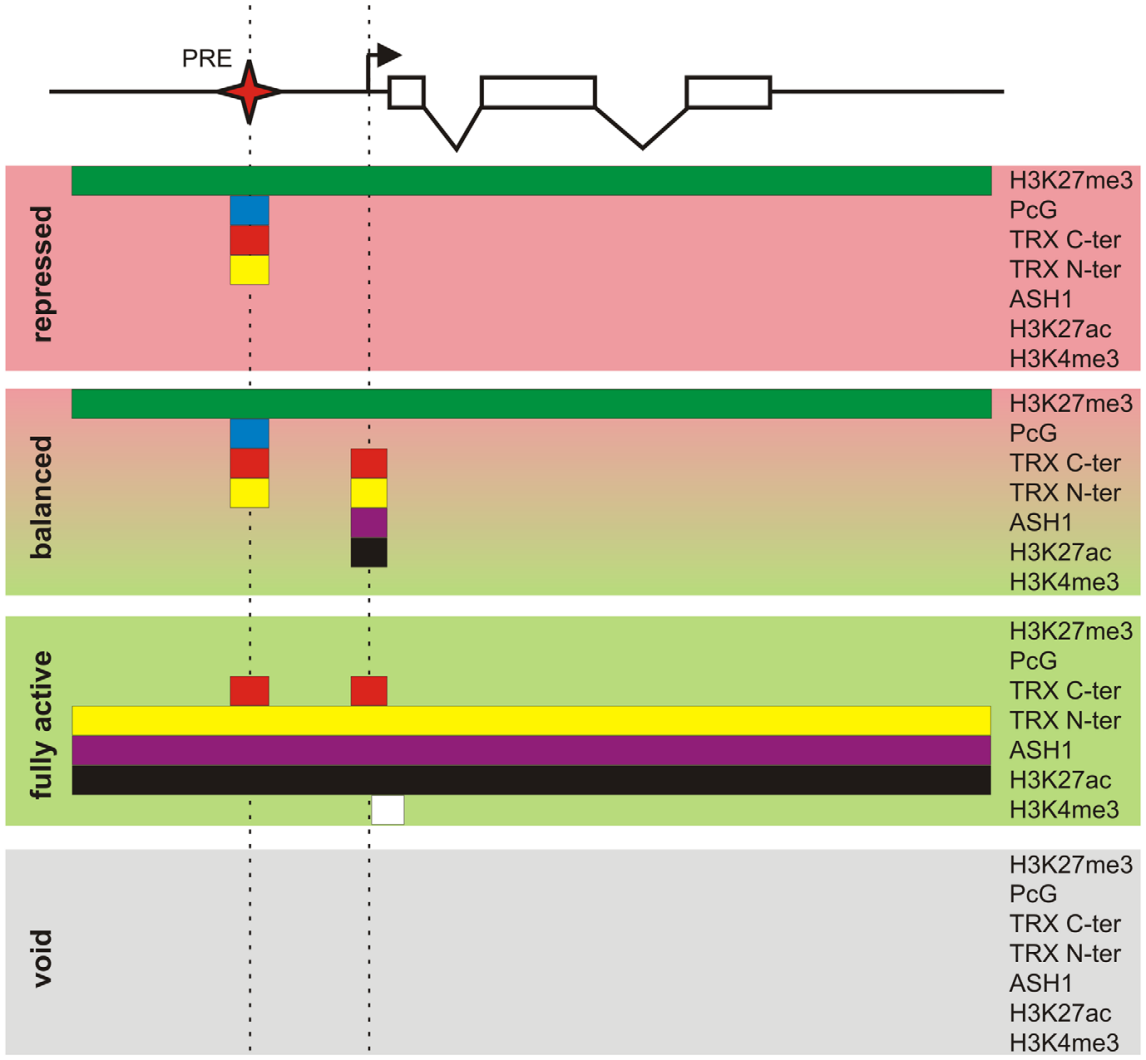
Protein landscapes of alternative chromatin states. A generic Polycomb target gene represented by a series of exons (open boxes) and introns (broken lines) can be found in four alternative epigenetic chromatin states. The “repressed” state is characterized by a broad domain of H3K27me3 whose extent is marked by the green bar. In this state the PcG proteins, whose distribution is indicated by the blue bar, and both TRX C-ter (red bar) and TRX N-ter (yellow bar) are bound at the PRE (red star). The action of certain combinations of repressors and activators leads to the “balanced” chromatin state. This state is characterized by the presence of repressive PcG marks and binding of TRX C-ter, TRX N-ter, ASH1 (purple bar), and H3K27ac around the TSS (broken arrow). Massive influx of activators switches a target gene to the “fully active” state. In this state the PcG proteins no longer bind to the PRE but TRX C-ter still associates with the PRE and also with the active TSS. The broad H3K27me3 domain is replaced by a domain of H3K27ac, TRX N-ter, and ASH1. The strong transcription of a target gene is accompanied by trimethylation of H3K4 downstream of the TSS (white bar). An unknown sequence of events results in the “void” chromatin state in which a target gene has lost PcG and TrxG regulation but remains transcriptionally inactive.

Key to our current understanding of PcG mechanisms is the fact that, while PcG proteins are present in most kinds of cells, the decision whether or not to repress a target gene depends crucially on whether that gene had been repressed in the previous cell cycle. This effect is responsible for the epigenetic maintenance of the repressed state and associated chromatin modifications. Similarly, through the action of TRX and ASH1, a PcG target gene that had not been repressed tends not to become repressed in the subsequent cell cycle and remains susceptible to transcriptional activators. By comparing PcG/TrxG and transcriptional landscapes in three lines of Drosophila cultured cells we found that the full repertoire of chromatin states that PcG target genes can assume is not limited to the repressed state dominated by PcG mechanisms and the transcriptionally active state governed by TrxG proteins but in addition includes transcriptionally active “balanced” states subjected to simultaneous or at least rapidly alternating control by both PcG and TrxG proteins, and a transcriptionally inactive “void” state lacking both PcG and TrxG control (Figure 8). Thus, although PcG mechanisms first achieved fame for producing stable long-term silenced states in Drosophila homeotic genes, it is clear that, in the general case, PcG states are not necessarily stable nor long-term.

### Coupling of PcG and TrxG regulation

Our results establish clearly that in robust PcG target regions (i.e. Class I PcG target regions) PcG and TrxG regulation are tightly coupled. Considering the role of MLL1 in the regulation of HOX genes [28] and the similarity between PcG complexes in flies and mammals, we expect that the same holds true for mammalian cells. It is possible that PcG and TRX recruitment to PRE/TREs share some DNA-binding proteins or DNA motifs. It will be important to determine whether PcG and TRX bind simultaneously or alternate over time. The nature of the TRX complex that binds to PRE/TREs remains enigmatic. To date the only TRX complex characterized biochemically is TAC1, purified from *Drosophila* embryos [29]. It is said to contain uncleaved full length TRX, anti-phosphatase Sbf1 and histone acetyltransferase dCBP. We can detect no uncleaved TRX in the nuclei of cultured cells (Y.B.S., T.G.K. and V.P., unpublished) indicating that the TRX bound at PRE/TREs of repressed genes does not represent TAC1. Proteolytically cleaved human orthologs of TRX, MLL1 and MLL2 have been purified as part of complexes similar in composition to the yeast COMPASS [7]–[8],[14]. Although the PRE/TRE binds both parts of the cleaved TRX, it lacks some COMPASS components (Y.B.S., T.G.K. and V.P., unpublished) and lacks H3K4 trimethylation, suggesting that it involves a different complex whose composition is yet to be characterized.

### The characteristic features of transcriptionally active PcG target genes

Consistent with genetic evidence, the presence of ASH1 and TRX at PcG target regions is linked to their transcriptional activity. However the two proteins show important differences in their behavior: binding of ASH1 is limited to transcriptionally active (fully derepressed or balanced) PcG targets and is not detected at completely repressed target loci. TRX is more complex. Both N-ter and C-ter parts of the protein associate with PREs regardless of the transcriptional status of their target genes and bind in the vicinity of TSS specifically when a target gene is transcriptionally active. In addition, the N-ter moiety of TRX together with ASH1 forms broad domains that encompass transcriptionally active PcG target genes. The different behavior of N-ter and C-ter parts of TRX may account for the discrepancy between reports of the co-localization of TRX and PcG proteins at many chromosomal sites [30] or PREs [17],[18],[24],[25] and reports claiming that TRX binds exclusively to transcriptionally active target genes [31]–[33]. The different accounts are due to the use of anti-TRX antibodies specific to different parts of the protein.

Whether the C-ter or N-ter specific antibodies were used, the number of TRX bound regions detected in our experiments is small compared to the number of active genes. This argues against a general role for TRX in transcription and is consistent with the limited number of regions detected on polytene chromosomes by various antibodies directed against C-ter or N-ter TRX [30],[31]. In marked contrast to these observations, Schuettengruber et al. [34] have recently reported exclusive association of N-ter but not C-ter moiety of TRX with TSS of most active transcription units in the chromatin of embryonic cells. The same report also asserted that in embryonic cells TRX C-ter but not TRX N-ter is bound at PREs. Remarkably the TRX N-ter specific antibody used by Schuettengruber et al. [34] is reportedly the same as one of the two used in our experiments [31]. While we cannot exclude the possibility that the behavior of the N-ter moiety of TRX in embryos is totally different from that in cultured or salivary gland cells, we suspect that more likely the preparation of the antibody used by Schuettengruber et al. [34] cross-reacted with some general transcription factor particularly abundant in embryonic cells. This emphasizes the importance, even the necessity, of using two or more independent antibodies to verify genome-wide ChIP results.

The RNAi knockdown experiments show that the broad binding of ASH1 and TRX N-ter within transcriptionally active PcG target regions is interdependent. This is consistent with the reported dissociation of ASH1 from polytene chromosomes of the salivary gland cells subjected to TRX RNAi [32] and the severe reduction of TRX N-ter binding to polytene chromosomes of *ash1* mutant larvae [31]. Despite interdependence in binding there is no compelling evidence that ASH1 and TRX N-ter are in the same protein complex. Although an interaction between TRX and ASH1 has been reported, it was said to require the intact SET domain of TRX, which is absent from its N-ter moiety [35]. We have not found TRX N-ter and ASH1 to co-precipitate from nuclear extracts (K.O. and V.P. unpublished), strengthening the impression that the two peptides do not interact directly.

A histone mark associated with ASH1/TRX N-ter domains is H3K27ac. Acetylation of H3K27 can antagonize PcG activity by competing with the placement of the H3K27me3 mark, which in our model is needed for effective contact of the PRE complex with the promoter, as well as for stable PcG binding. In fact, targeting a histone H3 acetylase to a PRE is sufficient to prevent the epigenetic maintenance of repression [2]. A recent study by Tie et al. [36] indicates that in *Drosophila* the HAT responsible for bulk acetylation of H3K27 is CREB-binding protein (dCBP), encoded by the *nejire* gene. Direct association of both TRX and ASH1 with dCBP was previously reported [29],[37]. Consistent with this, the TRX knock-down experiment, which also impairs ASH1 binding, shows that either or both proteins promote acetylation of K27 in the chromatin of active PcG targets. It also shows, however, that the level of H3K27ac is not directly related to the amount of ASH1 or TRX N-ter bound. It is possible that a small amount of TRX and/or ASH1 remaining on the chromosomes after RNAi depletion is sufficient to target enough HAT activity to maintain nearly normal levels of H3K27ac. Alternatively H3K27 acetylation may be produced by a process that is not mechanistically linked to ASH1 or TRX but is promoted by the two. A global reduction of immunostaining of polytene chromosomes with anti-H3K27ac antibodies and a global elevation of immunostaining with anti-H3K27me3 antibodies in *trx* mutant larvae was recently reported [36],[33]. We did not detect any global changes in either H3K27ac or H3K27me3 levels in our experiments. It is possible that the effects are generally weak and could only be detected on polytene chromosomes that consist of thousands of chromatin fibers bundled together. We note, however, that the reported changes of H3K27 acetylation and trimethylation levels involved numerous chromosomal sites, most of which, according to our data, do not stably bind PcG or TrxG, suggesting that in these cases the effect of *trx* mutation may have been indirect.

Our microarray data show that narrower peaks of H3K27ac are also found near numerous active promoters, at genes not known to be PcG targets. We speculate that a role of H3K27ac at these promoters may be to antagonize dimethylation of H3K27, which is abundantly distributed throughout the genome [38] and may have a general negative effect on transcription.

### The “balanced” chromatin state

In *Drosophila* cultured cells most PcG target genes are either completely repressed or fully derepressed or entirely devoid of both PcG and TrxG regulation. However in about 5% of cases, exemplified by the *Psc*-*Su(z)2* or *inv*-*en* loci, we see that binding of PcG complexes does not result in complete transcriptional silencing and can coexist with binding of ASH1/TRX N-ter. Whether a PcG target gene is capable of and will assume a “balanced” state may depend on the nature of the PRE, its binding complexes, and the promoter of the target gene. More likely, however, the major determinants are the nature and concentration of activators and repressors that act in concert with PcG/TrxG. Consistent with this idea, in imaginal disc cells, which are controlled by much more complex regulatory networks than cultured cells, simultaneous presence of both PcG and TrxG proteins at the transcriptionally active PcG target genes appears to be more common [18],[39]. Interestingly a common feature of the “balanced” chromatin state in both cultured and imaginal disc cells is the confinement of ASH1/TRX N-ter binding to the regions immediately around the promoters. We take this as a hint that the formation of a broad ASH1/TRX N-ter domain starts in the vicinity of the TSS.

Much has been said about the “bivalent” state, i.e. containing both PcG repressive marks and transcriptional activity marks, as characteristic of genes in mammalian embryonic stem cells [22]. In *Drosophila*, in cases such as those of the *Psc*-*Su(z)2* or *inv*-*en* loci, the balanced action of PcG and TrxG results in chromatin states similar in appearance to the “bivalent” state. We suppose that, like the “balanced” chromatin state of *Drosophila* PcG targets, the “bivalent” domains of embryonic stem cells would be associated with both PcG proteins and mammalian orthologs of TRX and ASH1.

### The “void” chromatin state

PcG target genes may also assume a state lacking both PcG and TrxG proteins. The fact that in several instances the same locus resides in this “void” chromatin state in cultured cells of completely different origin argues against it being a product of genomic aberrations. Several experimental observations also indicate that the “void” state is not a peculiarity of cultured cells. Thus, in salivary glands the *hh* gene lacks PcG binding and H3K27me3 but remains transcriptionally inactive [40], as in D23 cells. More recently a comparison of embryonic and imaginal disc cells showed that in many cases lack of PC binding was not accompanied by transcriptional activity [41]. The void chromatin state might be simply interpreted as a derepressed region that is transcriptionally inactive because the needed activator is absent. However, this does not explain why in this state TRX is also absent from the PRE, implying that neither PcG nor TRX binding is the default state of the PRE or that some specific condition prevents the recruitment of both. We have not detected other known repressive marks such as H3K9 methylation at these sites (unpublished observations).

### Switching the chromatin states

In line with the finding that the lack of PcG repression in the “void” chromatin state does not automatically lead to the activation of a target gene is the observation that PC knock-down elicits a very specific genomic response. Remarkably *Sex combs reduced* (*Scr*), *Antennapedia* (*Antp*) and *Abd-B*, the HOX genes whose derepression in heterozygous *+/Pc* ^−^ flies gives the famous Polycomb phenotype, are also among the genes most sensitive to PC knockdown in BG3 cells. The sensitivity of these genes cannot be explained by intrinsically poor recruitment of PcG proteins as *Abd-B* and *Scr* are controlled by multiple strong PREs capable of robust repression when placed next to a reporter gene [42]–[43]. We suppose that the reason for the differential sensitivity to PC levels lies in the availability of the corresponding transcriptional activators. We propose that in the BG3 cells the transcriptional activators of sensitive PcG target genes are present but at levels insufficient to override repression under normal conditions. The knockdown of PC lowers the threshold required for derepression. We suggest that a general role of PcG and TrxG mechanisms is to modulate the constraints on the levels of transcriptional activators required to switch the expression of PcG target genes. This concept helps to explain why, despite the implication of the PcG system in the control of all morphogenetic pathways, the reduction of PcG levels during differentiation of mammalian cell lineages [44] or tissue regeneration in flies [45] results in the execution of very specific genomic programs.

## Materials and Methods

### Cell culture, chromatin immunoprecipitation, and microarray analysis

The description of cell lines and culturing conditions are detailed in Text S1. The source, amount and specificities of antibodies used for ChIP are indicated in Figure S11, Figure S12, and Table S9. ChIP, hybridization to *Drosophila* tiling arrays v1.0 (Forward) (Cat# 900587; Affymetrix) and primary data processing were done as described ([19], Text S1). To derive the genomic binding profile for a given protein, microarray hybridizations of the DNA from at least two independent ChIP experiments and two matching chromatin inputs were used (Table S10). The results were visualized with the Integrated Genome Browser (Affymetrix). Details of computational definitions of bound regions and comparisons of data sets are described in Text S1 and Table S12. For all analyses *D. melanogaster* Apr. 2004 (dm2) genome assembly and genome annotation version 4.3 were used.

### qRT–PCR and RNAi

Total RNA from 5×10^6^ cells was isolated using Trizol (Invitrogen) and 3 µg were used for random primed synthesis of cDNA with First Strand Synthesis Kit (GE Healthcare). The control reaction, omitting reverse transcriptase, was always run in parallel. cDNA was purified with QIAquick PCR Purification Kit (Qiagen) and eluted with 100 µl of elution buffer. qPCR was done with the Mx3000P instrument (Stratagene) in total volume of 10 µl containing 2.5 µl of cDNA solution, 1xSYBR Green PCR Master Mix (ABgene), 100 nM of corresponding primers and 100 nM of ROX as a reference dye. The sequences of primers are given in Table S11. Serial dilutions of genomic DNA were used to make the standard curve. The amount of cDNA for a gene of interest in the given preparation was expressed as a fraction of *RpL32* cDNA. The RNAi was performed as described [46] with minor modifications detailed in Text S1.

### Accession numbers

Microarray data are available from GEO under accession numbers GSE18100, GSE18176 and GSE18177.

## Supporting Information

Figure S1 Binding of H3K27me3 to Class II PcG target regions is generally weaker and more narrow than in Class I target regions. For each Class I (blue) and Class II (pink) target region the average extent of H3K27me3 enrichment (A, C, E) and the length of methylation domain (B, D, F) were computed and the density of observations plotted. The smoothed density estimates were plotted as dashed lines.
(1.63 MB TIF)

Figure S2 Changes in chromatin states of PcG target regions among cell lines. (A) Comparison of Class I PcG target regions with the absolute number of changes from one cell line to another as indicated. Grey indicates sites that do not change, white sites that lose E(Z) binding, black sites that lose all PcG binding. (B) Venn diagram representation of the overlap between Class I PcG target regions in Sg4 (red), BG3 (green), and D23 (blue) cell lines. The total number of sites (in parentheses) and the number of common regions for each cell line is indicated.
(0.66 MB TIF)

Figure S3 All cell lines contribute to the non-redundant catalogue of PcG target genes. The changes in the numbers of high-confidence (red line), low-confidence (blue line), and grand total number (green line) of PcG target genes were monitored as the data set (x-axis) from an additional cell line was considered. Each panel represents different sequence of comparisons indicated above the graphs. Note that irrespective of the direction of comparison the total number of targets (green line) continues to grow indicating that more potential PcG targets are yet to be discovered.
(0.21 MB TIF)

Figure S4 Binding of H3K4me3 to transcription units peaks at position +500 and in combination with binding of Pol II at TSS indicates transcriptional activity. (A) H3K4me3/Input ratios from 10 kb windows centered on TSS of transcription units bound by Pol II and H3K4me3 were collected and superimposed into a single scatter plot. The color (red = zero, white = highest) indicates the density of observations. The binding of H3K4me3 within a transcription unit is most prominent around position +500 (vertical dashed line). The weaker peak of H3K4me3 upstream of TSS is due to a high number of closely juxtaposed divergently transcribed genes. (B) The expression of eight randomly chosen transcription units from a group that binds Pol II at TSS and H3K4me3 around position +500 in Sg4 cells (red bars) was compared to expression of eight randomly chosen transcription units that show no binding of these proteins in Sg4 cells (blue bars). The abundance of transcripts in the total mRNA pool from Sg4 cells was assayed by qRT-PCR and normalized to the abundance of *RpL32* transcript. The mean and the scatter (error bars) from the two independent experiments is shown. Note the log10 scale of the y-axis. The Wilcoxon rank sum test shows that the difference in the expression levels between the two groups is statistically significant (W = 64; p-value = 7.77e-05).
(1.39 MB TIF)

Figure S5 *Bithorax-Complex* in D23 cells. The results of ChIP-chip experiments with antibodies indicated on the right of each graph were expressed as smoothed ChIP/Input signal ratios averaged for two independent experiments and plotted along *Drosophila* release 2004 coordinates. The positions and the exon structure of annotated transcripts are shown above (transcription left to right) and below (transcription right to left) the coordinate scale (in bp). As is evident from the presence of PC and H3K27me3 and complete absence of Pol II and H3K4me3, all genes of the BX-C are fully repressed in this cell line.
(0.20 MB TIF)

Figure S6 Knock-down of PC and ASH1 by RNAi. To estimate the efficiency of RNAi knock-downs serial dilutions of total nuclear protein from cells treated with dsRNA specific to the protein of interest were separated by SDS-PAGE and transferred to the membrane alongside with equal amounts of total nuclear protein from cells treated with mock (LacZ) dsRNA. Western blots were stained with anti-PC (A) and anti-ASH1 (B) antibodies and the intensity of the signals compared. The amounts of nuclear protein loaded (in µl) are indicated above each lane. The leftmost lane of each western blot shows the migration of molecular weight standards (in kDa). (C) Coomassie staining of SDS-PAGE of equal amounts of total nuclear protein from corresponding cells (indicated above the lanes) served as loading control.
(1.83 MB TIF)

Figure S7 The gene *tiptop* can assume a “void” state. The *tiptop* gene is PcG-silenced in BG3 cells (B) with PC and TRX bound at PRE (blue shade) but is in the “void” state in Sg4 cells (A), with no PC and H3K27me3 but also lacking TRX, ASH1, Pol II, and H3K4me3.
(0.18 MB TIF)

Figure S8 The distribution of H3K9ac within and outside ASH1/TRX N-ter domains is similar. The TSS of active transcription units within (A) and outside ASH1/TRX N-ter domains (B) were defined based on Pol II and H3K4me3 binding. The log_2_-transformed H3K9ac/Input ratios from 10 kb windows centered on these TSS were collected and superimposed into a single scatter plot. The color (red = zero, white = highest) indicates the density of observations. Both within and outside ASH1/TRX N-ter domains the distribution of H3K9ac peaks around position +450 downstream of the TSS (vertical dashed line).
(0.16 MB TIF)

Figure S9 The *en-inv* locus in D23 cells. In these cells the locus resides in fully repressed state as judged by the presence of H3K27me3, PC and E(Z) (not shown) and complete absence of H3K4me3 and Pol II.
(0.13 MB TIF)

Figure S10 The expression of *mirr* gene after various RNAi treatments. The histogram shows the mean of two independent qRT-PCR experiments with error bars indicating the scatter.
(0.22 MB TIF)

Figure S11 The binding sites recognized by both rabbit polyclonal and rat monoclonal anti-ASH1 antibodies include all the broadest and strongest ASH1 binding regions. The set of ASH1 bound regions detected in Sg4 cells with rabbit polyclonal antibodies was compared to the set of regions detected in these cells with rat monoclonal antibodies. The length (B) and the extent of ASH1 binding (A) within the regions unique to the polyclonal antibody data set (empty bars) and the regions recognized by both antibodies (red bars) were computed and the frequencies of observations plotted. The extent of ASH1 binding detected with polyclonal antibodies was defined as average smoothed IP/Input ratio for the six consecutive features which showed the strongest binding within the bound region. The reciprocal comparison and the comparisons in BG3 and D23 cells gave the same results (data not shown).
(0.42 MB TIF)

Figure S12 Comparison of antibodies against TRX C-ter raised by Poux et al. (2002) and Beisel at al. (2007). The Venn diagrams illustrate overlapping between sets of regions detected by the two antibodies in Sg4 cells (A) within PcG target regions, (B) outside PcG target regions. The total number of regions in the group and percentage of overlapping are indicated.
(0.33 MB TIF)

Table S1 PcG target regions.
(0.02 MB XLS)

Table S2 Class I high-confidence PcG target genes.
(0.09 MB XLS)

Table S3 Class I low-confidence PcG target genes.
(0.07 MB XLS)

Table S4 Class II high-confidence PcG target genes.
(0.03 MB XLS)

Table S5 Class II low-confidence PcG target genes.
(0.05 MB XLS)

Table S6 List of computationally defined PREs.
(0.03 MB XLS)

Table S7 List of PcG target regions found in void state.
(0.02 MB XLS)

Table S8 List of Class I PcG target regions in “balanced” state.
(0.02 MB XLS)

Table S9 List of antibodies used.
(0.02 MB XLS)

Table S10 List of microarray data sets.
(0.03 MB XLS)

Table S11 List of oligonucleotide primers used in this work.
(0.02 MB XLS)

Table S12 List of Max Distance parameter values used in definition of bound regions.
(0.01 MB XLS)

Text S1 Supplemental notes.
(0.05 MB DOC)
