# Supplementary figures and images for: Alternative Epigenetic Chromatin States of Polycomb Target Genes
Source: PLoS Genet. 2010 Jan 8;6(1):e1000805. doi: 10.1371/journal.pgen.1000805 (PMC2799325; doi:10.1371/journal.pgen.1000805)

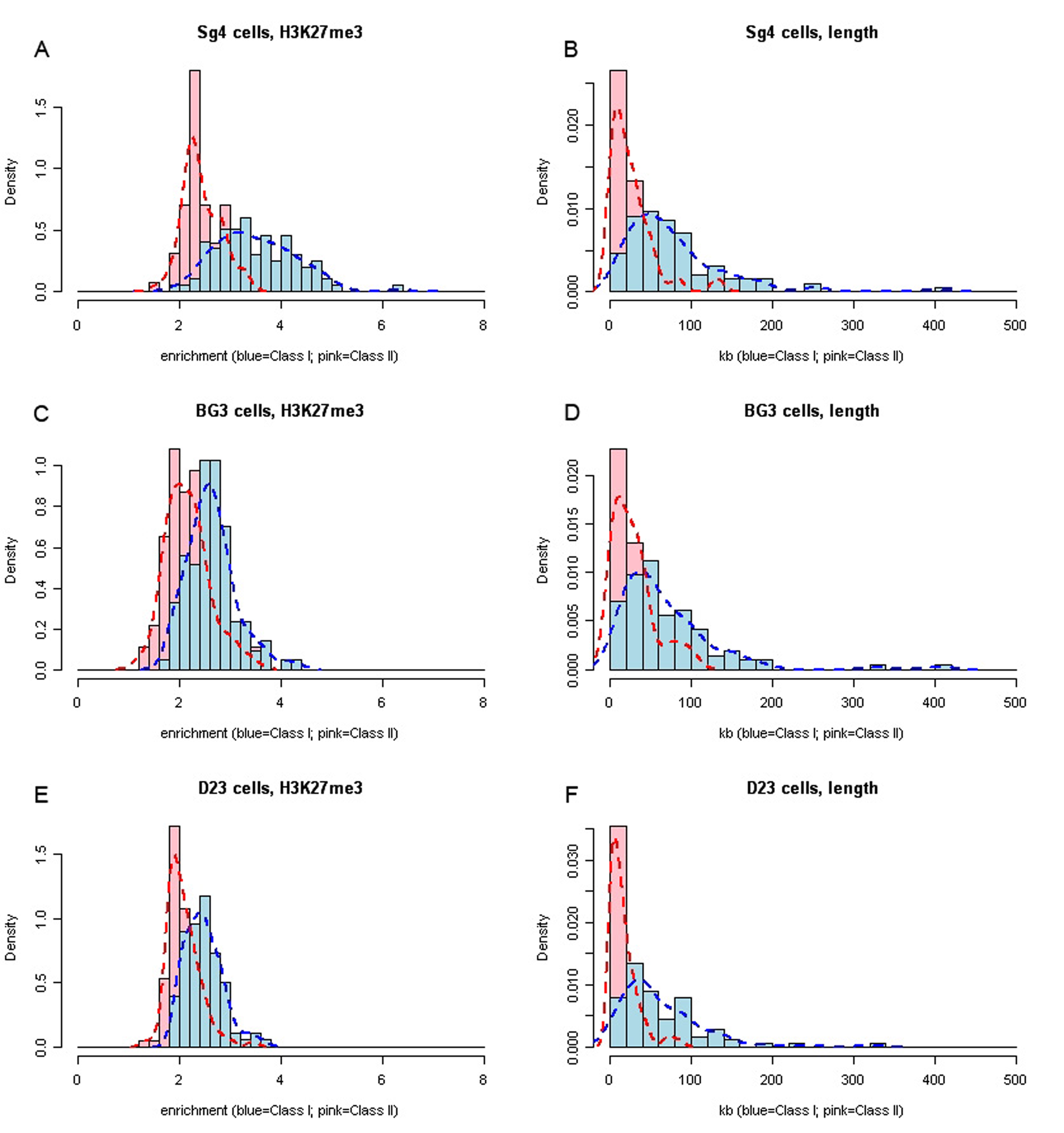

Supplement: Figure S1 — Binding of H3K27me3 to Class II PcG target regions is generally weaker and more narrow than in Class I target regions. For each Class I (blue) and Class II (pink) target region the average extent of H3K27me3 enrichment (A, C, E) and the length of methylation domain (B, D, F) were computed and the density of observations plotted. The smoothed density estimates were plotted as dashed lines. (1.63 MB TIF) [file pgen.1000805.s001.tif]

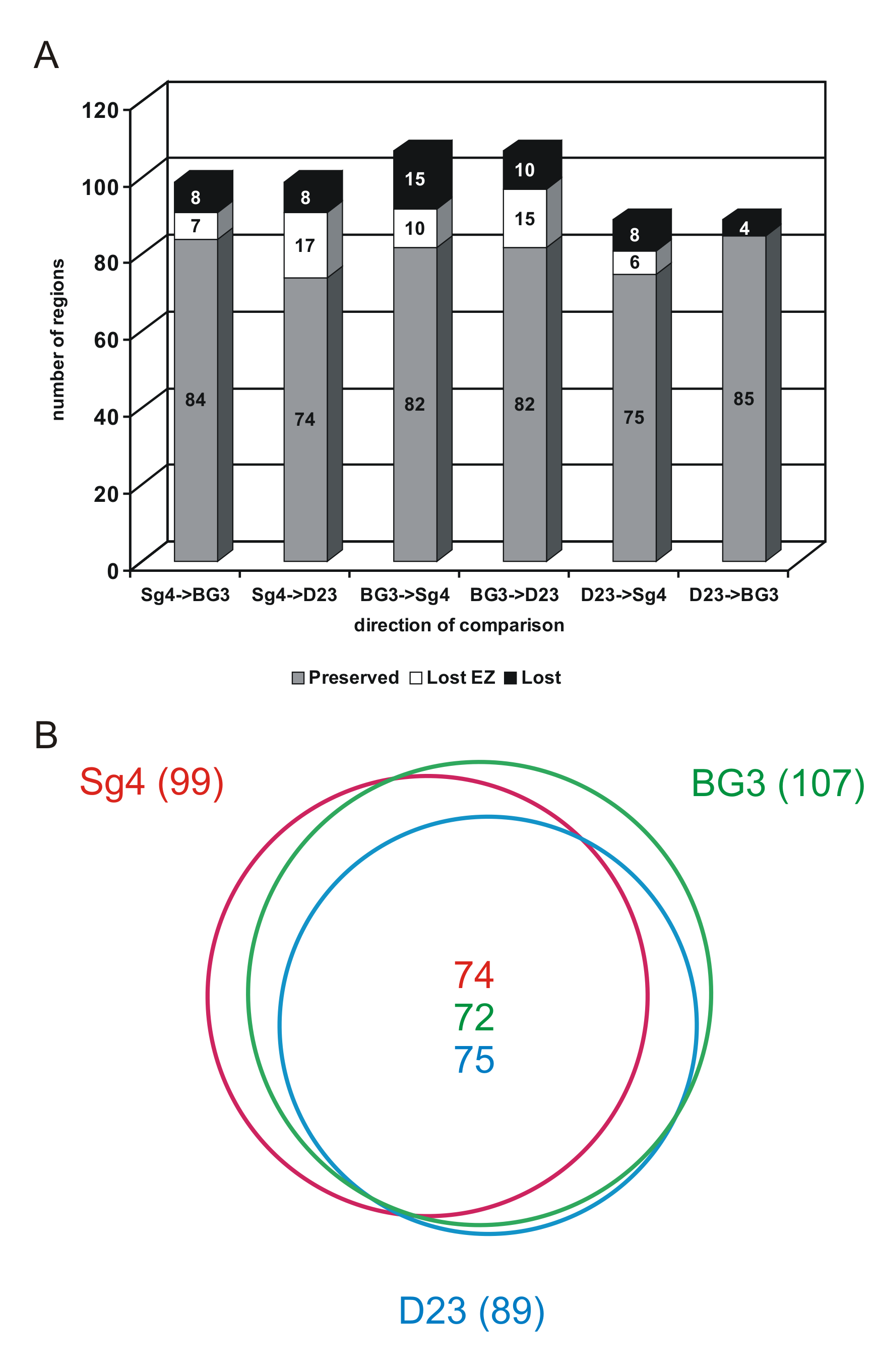

Supplement: Figure S2 — Changes in chromatin states of PcG target regions among cell lines. (A) Comparison of Class I PcG target regions with the absolute number of changes from one cell line to another as indicated. Grey indicates sites that do not change, white sites that lose E(Z) binding, black sites that lose all PcG binding. (B) Venn diagram representation of the overlap between Class I PcG target regions in Sg4 (red), BG3 (green), and D23 (blue) cell lines. The total number of sites (in parentheses) and the number of common regions for each cell line is indicated. (0.66 MB TIF) [file pgen.1000805.s002.tif]

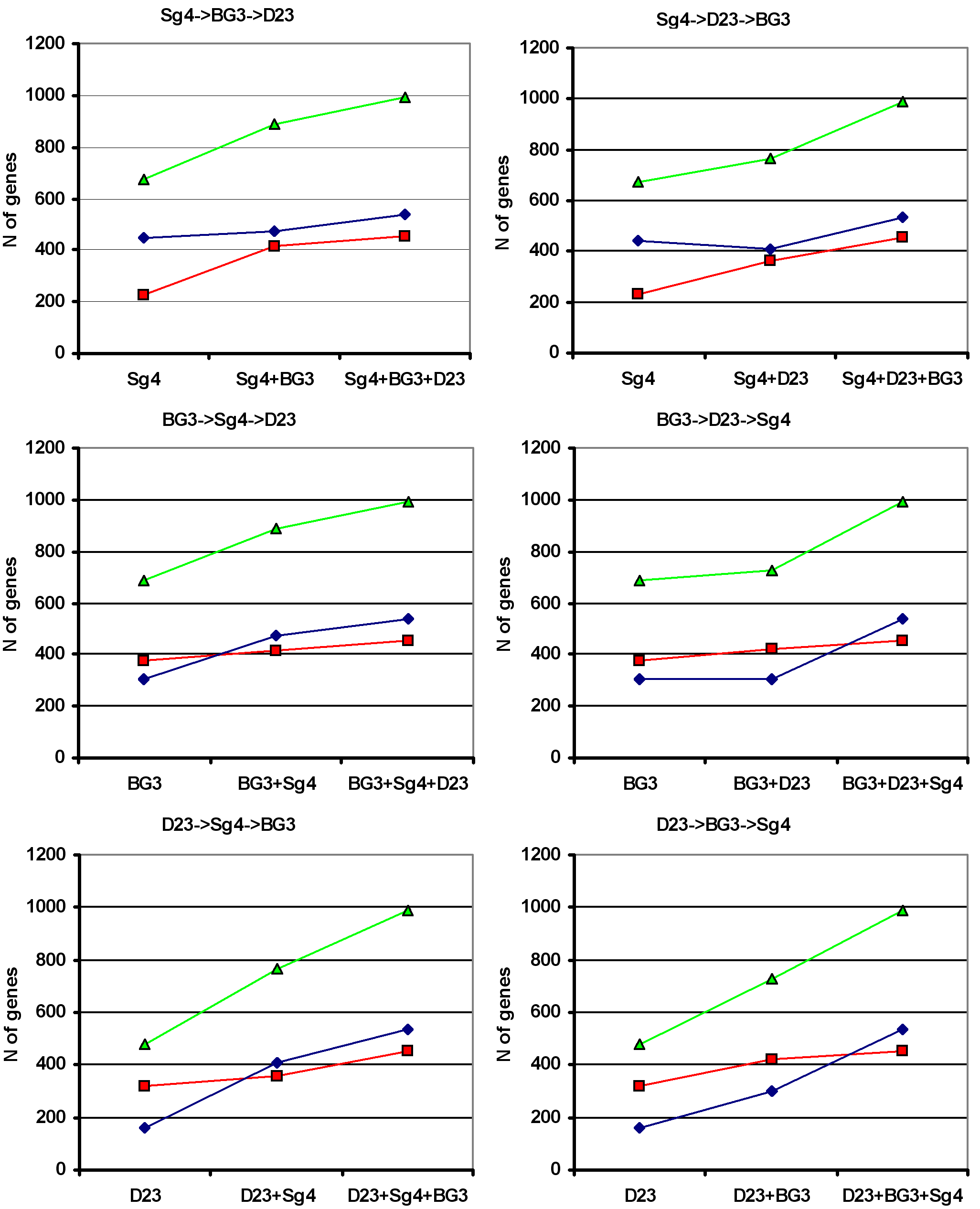

Supplement: Figure S3 — All cell lines contribute to the non-redundant catalogue of PcG target genes. The changes in the numbers of high-confidence (red line), low-confidence (blue line), and grand total number (green line) of PcG target genes were monitored as the data set (x-axis) from an additional cell line was considered. Each panel represents different sequence of comparisons indicated above the graphs. Note that irrespective of the direction of comparison the total number of targets (green line) continues to grow indicating that more potential PcG targets are yet to be discovered. (0.21 MB TIF) [file pgen.1000805.s003.tif]

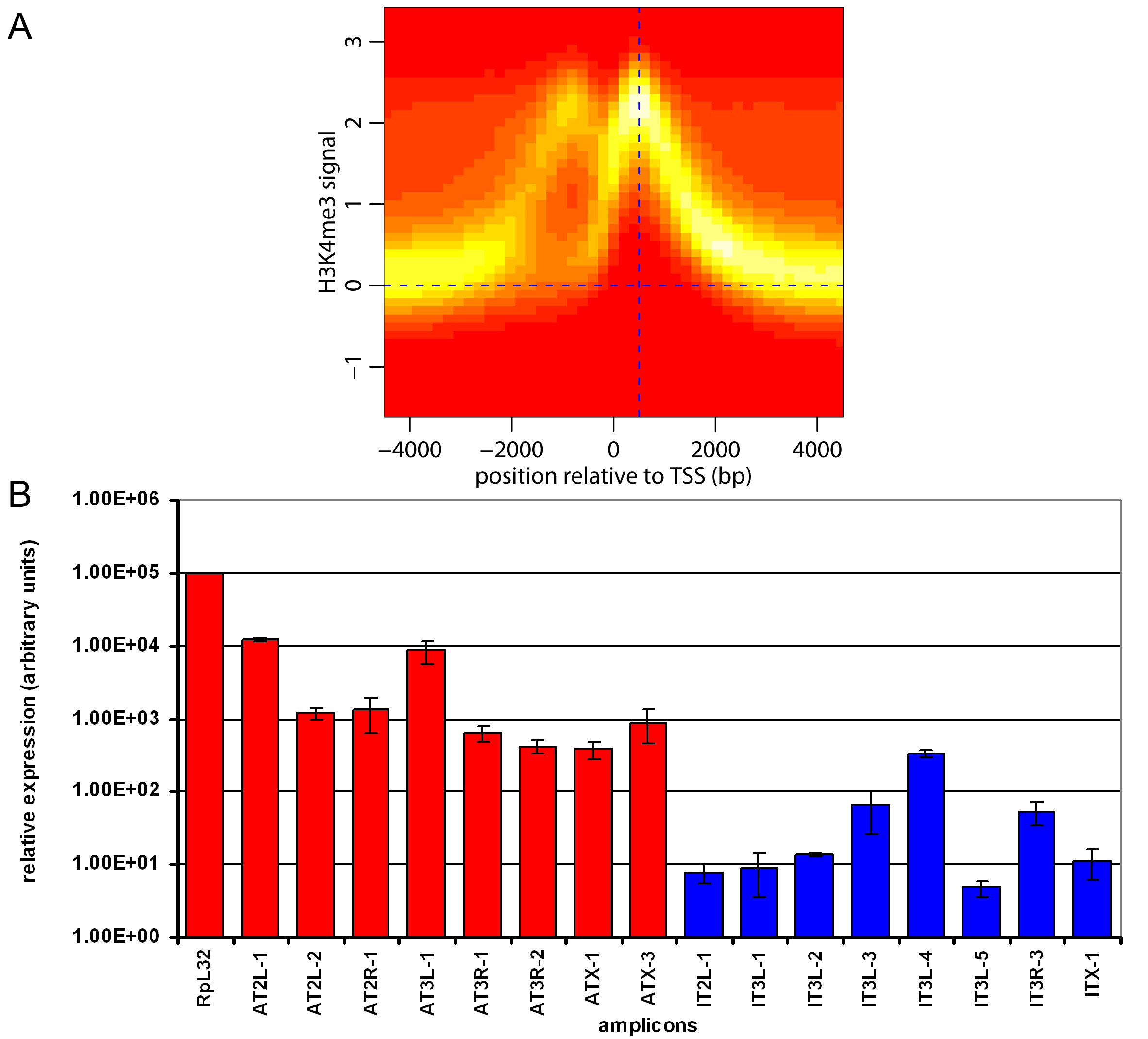

Supplement: Figure S4 — Binding of H3K4me3 to transcription units peaks at position +500 and in combination with binding of Pol II at TSS indicates transcriptional activity. (A) H3K4me3/Input ratios from 10 kb windows centered on TSS of transcription units bound by Pol II and H3K4me3 were collected and superimposed into a single scatter plot. The color (red = zero, white = highest) indicates the density of observations. The binding of H3K4me3 within a transcription unit is most prominent around position +500 (vertical dashed line). The weaker peak of H3K4me3 upstream of TSS is due to a high number of closely juxtaposed divergently transcribed genes. (B) The expression of eight randomly chosen transcription units from a group that binds Pol II at TSS and H3K4me3 around position +500 in Sg4 cells (red bars) was compared to expression of eight randomly chosen transcription units that show no binding of these proteins in Sg4 cells (blue bars). The abundance of transcripts in the total mRNA pool from Sg4 cells was assayed by qRT-PCR and normalized to the abundance of RpL32 transcript. The mean and the scatter (error bars) from the two independent experiments is shown. Note the log10 scale of the y-axis. The Wilcoxon rank sum test shows that the difference in the expression levels between the two groups is statistically significant (W = 64; p-value = 7.77e-05). (1.39 MB TIF) [file pgen.1000805.s004.tif]

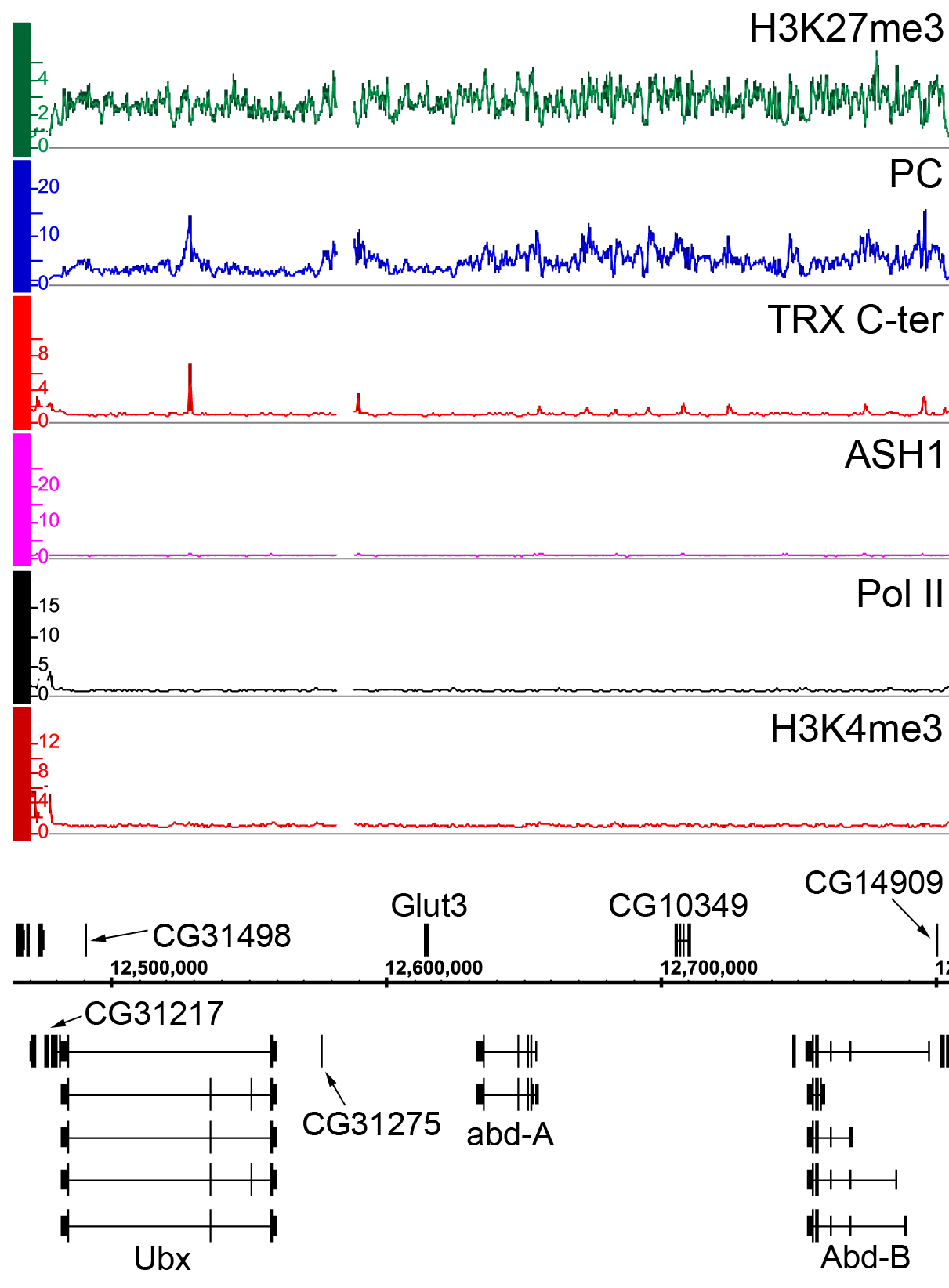

Supplement: Figure S5 — Bithorax-Complex in D23 cells. The results of ChIP-chip experiments with antibodies indicated on the right of each graph were expressed as smoothed ChIP/Input signal ratios averaged for two independent experiments and plotted along Drosophila release 2004 coordinates. The positions and the exon structure of annotated transcripts are shown above (transcription left to right) and below (transcription right to left) the coordinate scale (in bp). As is evident from the presence of PC and H3K27me3 and complete absence of Pol II and H3K4me3, all genes of the BX-C are fully repressed in this cell line. (0.20 MB TIF) [file pgen.1000805.s005.tif]

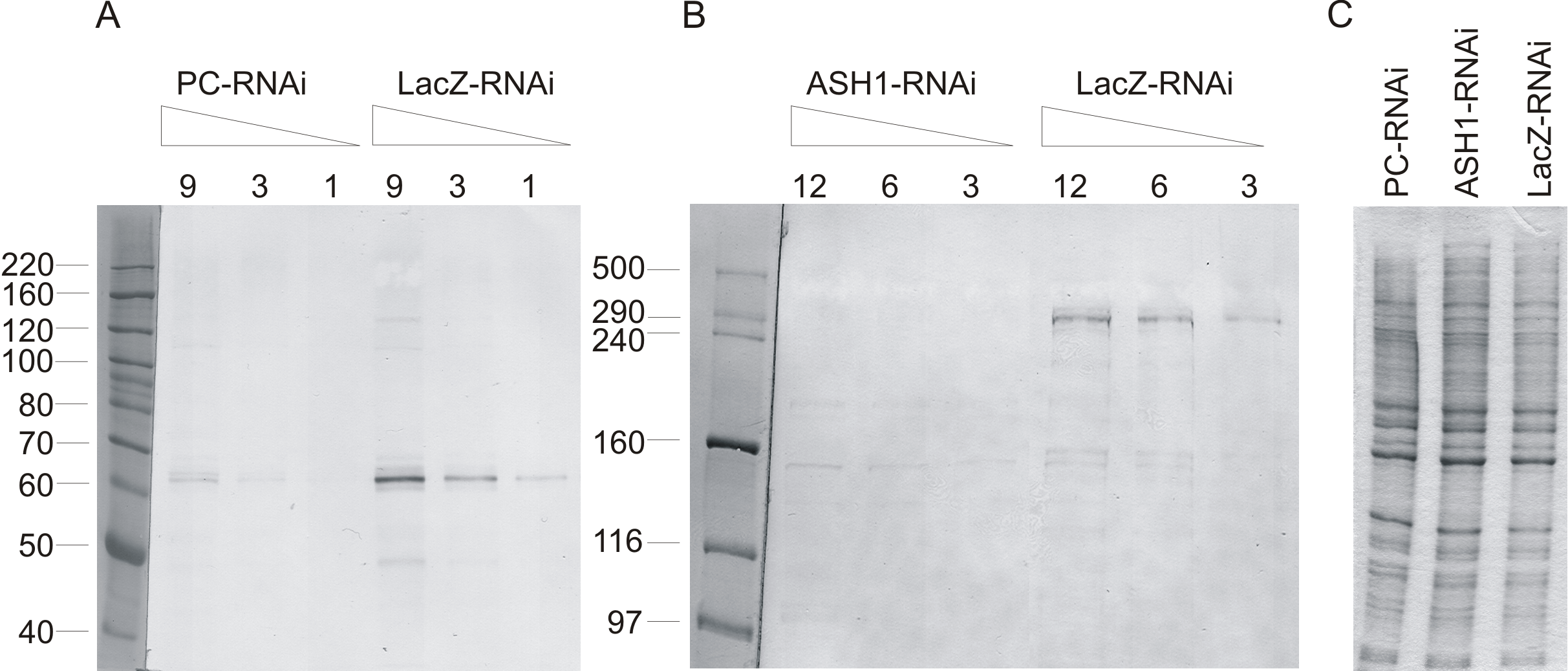

Supplement: Figure S6 — Knock-down of PC and ASH1 by RNAi. To estimate the efficiency of RNAi knock-downs serial dilutions of total nuclear protein from cells treated with dsRNA specific to the protein of interest were separated by SDS-PAGE and transferred to the membrane alongside with equal amounts of total nuclear protein from cells treated with mock (LacZ) dsRNA. Western blots were stained with anti-PC (A) and anti-ASH1 (B) antibodies and the intensity of the signals compared. The amounts of nuclear protein loaded (in µl) are indicated above each lane. The leftmost lane of each western blot shows the migration of molecular weight standards (in kDa). (C) Coomassie staining of SDS-PAGE of equal amounts of total nuclear protein from corresponding cells (indicated above the lanes) served as loading control. (1.83 MB TIF) [file pgen.1000805.s006.tif]

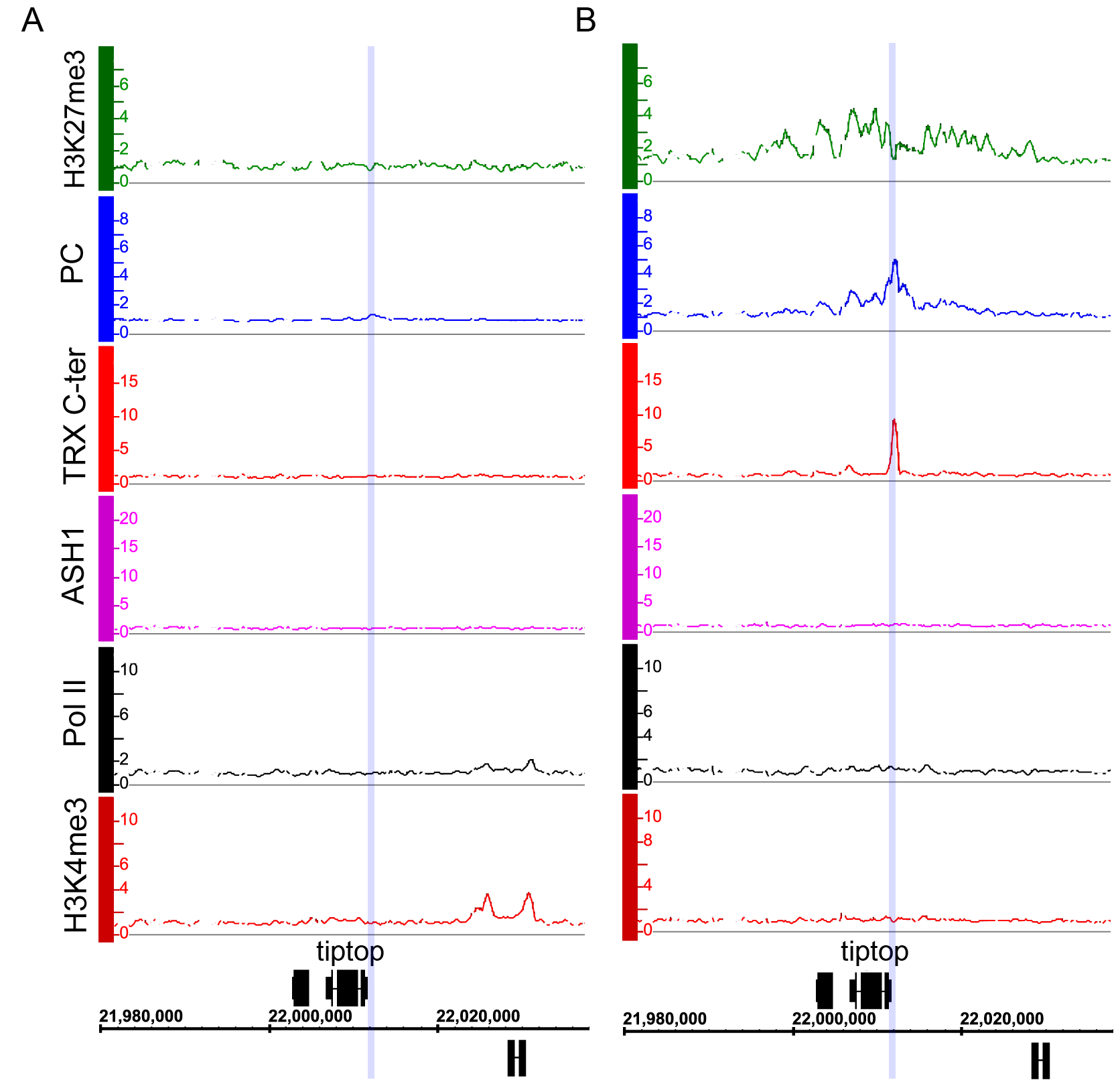

Supplement: Figure S7 — The gene tiptop can assume a “void” state. The tiptop gene is PcG-silenced in BG3 cells (B) with PC and TRX bound at PRE (blue shade) but is in the “void” state in Sg4 cells (A), with no PC and H3K27me3 but also lacking TRX, ASH1, Pol II, and H3K4me3. (0.18 MB TIF) [file pgen.1000805.s007.tif]

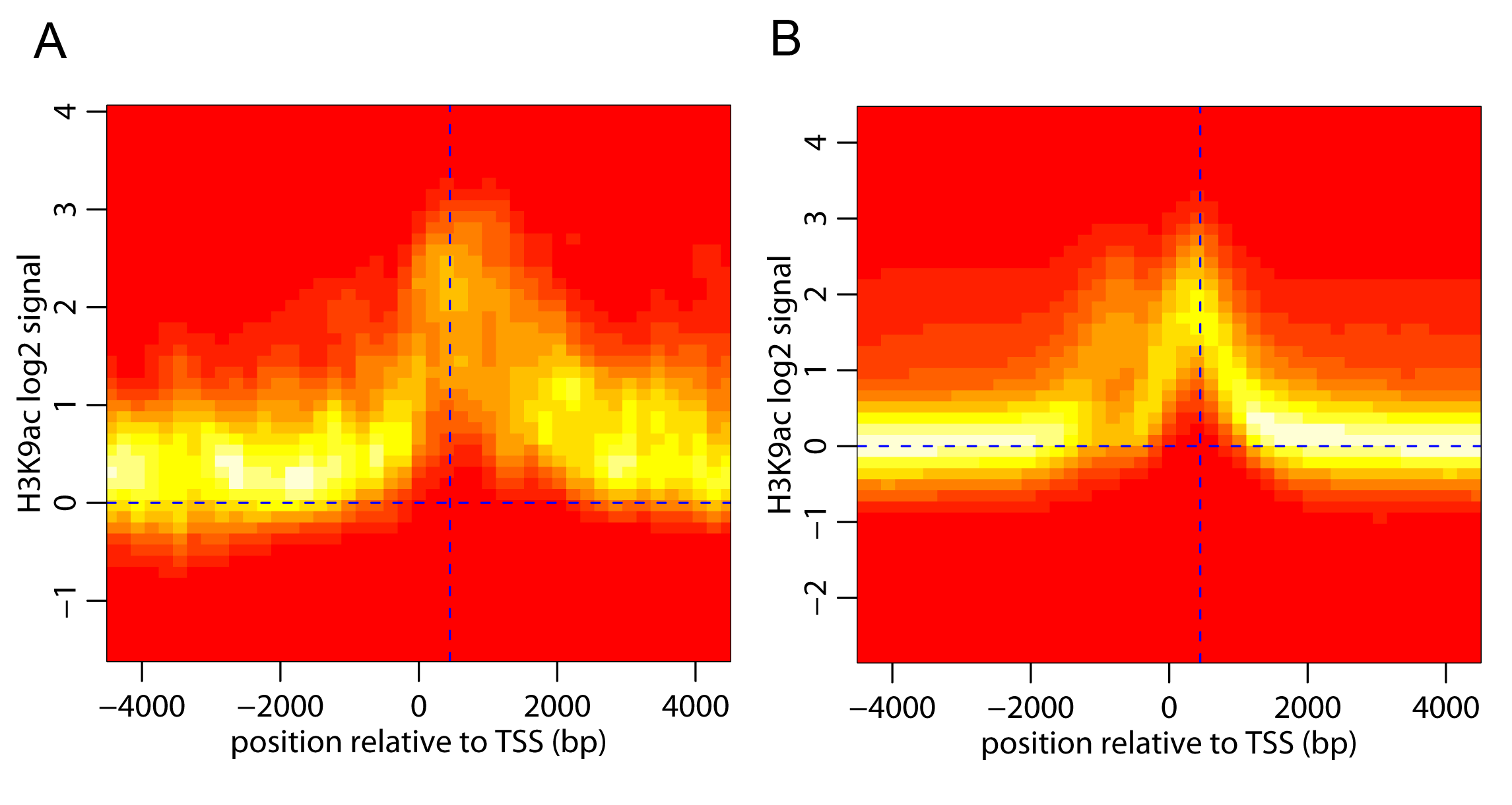

Supplement: Figure S8 — The distribution of H3K9ac within and outside ASH1/TRX N-ter domains is similar. The TSS of active transcription units within (A) and outside ASH1/TRX N-ter domains (B) were defined based on Pol II and H3K4me3 binding. The log2-transformed H3K9ac/Input ratios from 10 kb windows centered on these TSS were collected and superimposed into a single scatter plot. The color (red = zero, white = highest) indicates the density of observations. Both within and outside ASH1/TRX N-ter domains the distribution of H3K9ac peaks around position +450 downstream of the TSS (vertical dashed line). (0.16 MB TIF) [file pgen.1000805.s008.tif]

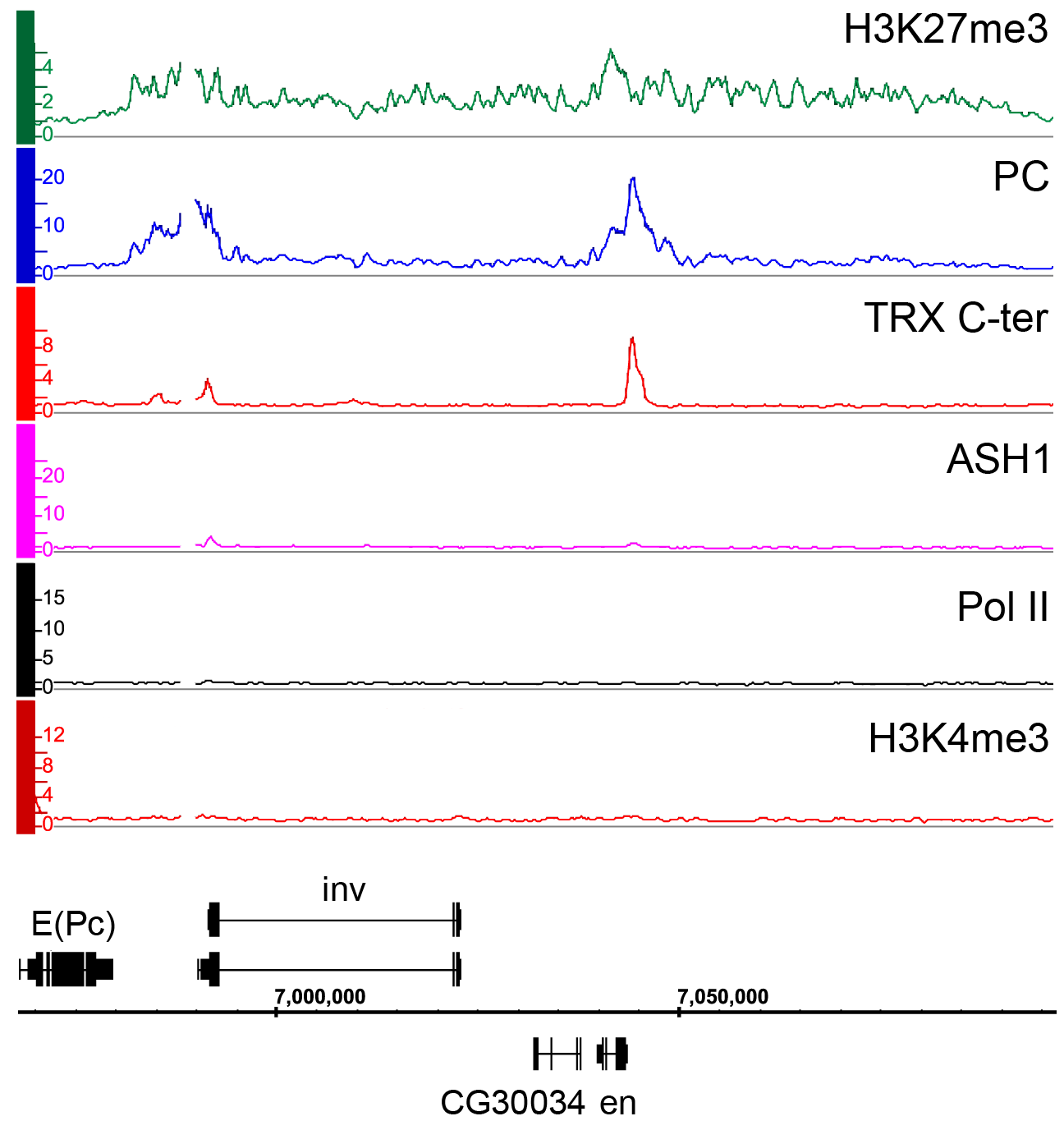

Supplement: Figure S9 — The en-inv locus in D23 cells. In these cells the locus resides in fully repressed state as judged by the presence of H3K27me3, PC and E(Z) (not shown) and complete absence of H3K4me3 and Pol II. (0.13 MB TIF) [file pgen.1000805.s009.tif]

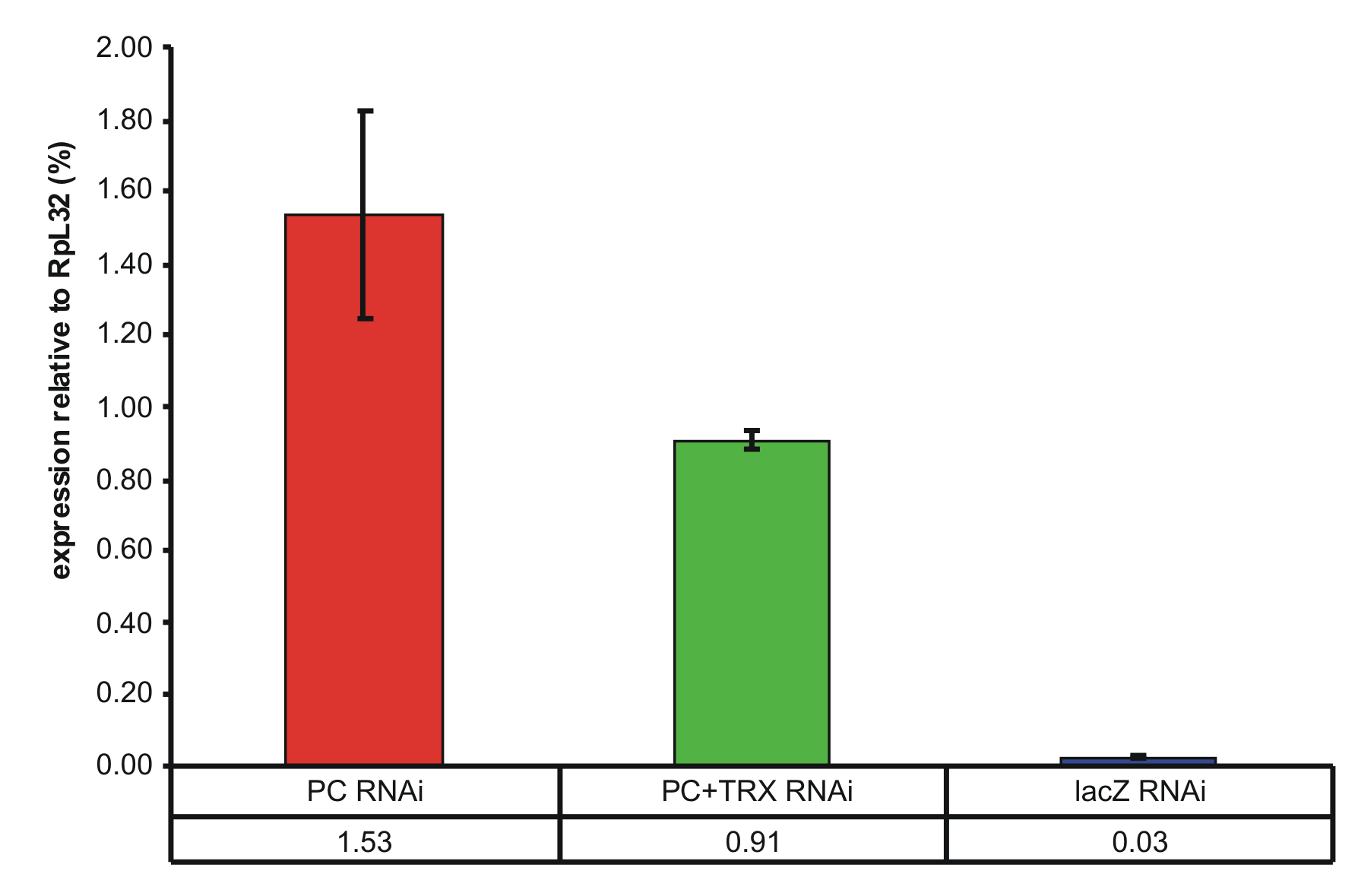

Supplement: Figure S10 — The expression of mirr gene after various RNAi treatments. The histogram shows the mean of two independent qRT-PCR experiments with error bars indicating the scatter. (0.22 MB TIF) [file pgen.1000805.s010.tif]

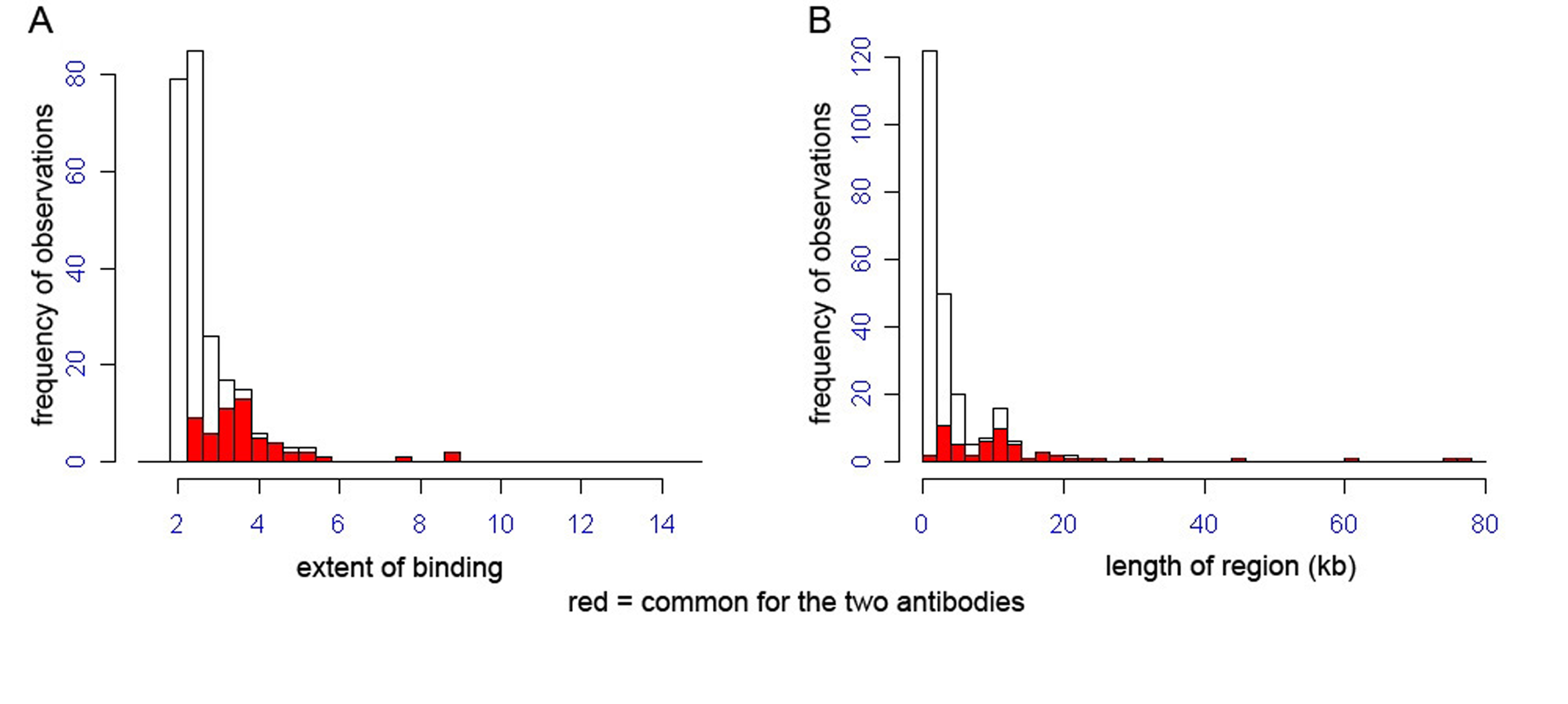

Supplement: Figure S11 — The binding sites recognized by both rabbit polyclonal and rat monoclonal anti-ASH1 antibodies include all the broadest and strongest ASH1 binding regions. The set of ASH1 bound regions detected in Sg4 cells with rabbit polyclonal antibodies was compared to the set of regions detected in these cells with rat monoclonal antibodies. The length (B) and the extent of ASH1 binding (A) within the regions unique to the polyclonal antibody data set (empty bars) and the regions recognized by both antibodies (red bars) were computed and the frequencies of observations plotted. The extent of ASH1 binding detected with polyclonal antibodies was defined as average smoothed IP/Input ratio for the six consecutive features which showed the strongest binding within the bound region. The reciprocal comparison and the comparisons in BG3 and D23 cells gave the same results (data not shown). (0.42 MB TIF) [file pgen.1000805.s011.tif]

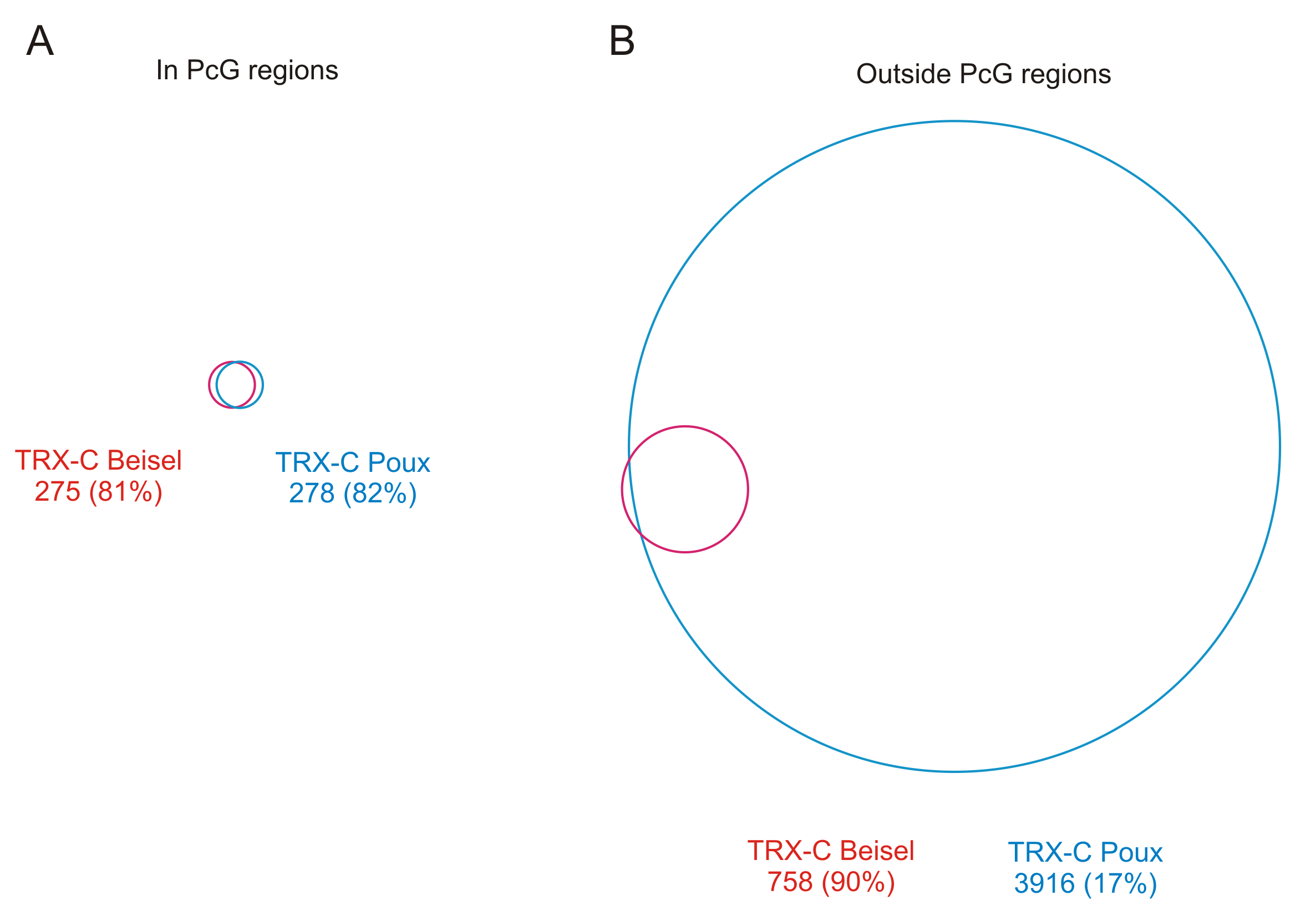

Supplement: Figure S12 — Comparison of antibodies against TRX C-ter raised by Poux et al. (2002) and Beisel at al. (2007). The Venn diagrams illustrate overlapping between sets of regions detected by the two antibodies in Sg4 cells (A) within PcG target regions, (B) outside PcG target regions. The total number of regions in the group and percentage of overlapping are indicated. (0.33 MB TIF) [file pgen.1000805.s012.tif]
