# Supplementary material for: Alternative Epigenetic Chromatin States of Polycomb Target Genes
Source: PLoS Genet. 2010 Jan 8;6(1):e1000805. doi: 10.1371/journal.pgen.1000805 (PMC2799325; doi:10.1371/journal.pgen.1000805)
Supplement: Text S1 — Supplemental notes. (0.05 MB DOC) [file pgen.1000805.s025.doc]

**Supplemental Notes – Text S1**

**High- and low- confidence PcG target genes**

Previously we have presented evidence that H3K27me3 spreads from the PRE through transient looping of PcG complexes anchored to the element (Kahn et al., 2006). Such spreading of the methylation domain may continue for tens of kilobases unless prevented by a boundary element or by active transcriptional activity. It is conceivable that in the densely populated *Drosophila* genome some genes become included in the H3K27me3 domain simply due to their proximity to the primary PcG target gene and generic lack of transcriptional activity in a given cell type. For these genes no correlation between binding of TrxG proteins and their transcriptional activity is expected. Since including them in the comparative analysis is likely to introduce unnecessary noise, we sought a way to filter them out. The looping model predicts that ChIP would detect strong PcG presence at PREs as well as at transient contact sites along the methylation domain that would depend on the frequency of contacts and on the crosslinking efficiency of the protein component being assayed. Of all PcG proteins tested, the levels of interaction within the methylation domain were always highest in the case of PC protein itself, consistent with the idea that the PC chromodomain interacts with nucleosomes and is more likely to become crosslinked to them (Schwartz et al., 2006; Kahn et al., 2006). Since the repressive effect of PcG proteins on transcription is at least in part mediated by direct interference with transcription initiation (Dellino et al., 2004), we reasoned that the degree of PC detection within 1 kb of the Transcription Start Site (TSS) of a gene might serve as readout of the degree of regulation. Thus of all genes within H3K27me3 domains we considered those that showed binding of PC within 500bp of the TSS as high-confidence PcG targets and all others as low-confidence targets. In the analysis of the correlation between transcriptional activity of PcG target genes and binding of ASH1 and TRX only high-confidence PcG target genes were considered.

**Simultaneous presence of Pol II at TSS and H3K4me3 at position +500 is a general indicator of transcription**

To obtain a reliable way to distinguish transcriptionally active from inactive genes, we first made a genome-wide map of RNA pol II (Pol II), H3K4me3 and H3K36me3 distributions in Sg4 cells. Of 17125 transcripts that have non-identical TSS, 6590 (38%) have a peak of Pol II associated with their TSS. 88% of these also have robust peaks of H3K4me3, usually positioned around +500bp downstream of the TSS (Figure S4A), as has also been found in mammals and yeast (Pokholok et al., 2005; Barski et al., 2007). Consistent with the idea that trimethylation of H3K4 is a mark for the early stages of transcription elongation, 94% of the genes marked by simultaneous presence of Pol II at the TSS and H3K4me3 around position +500 are also enriched in H3K36me3 in the 3’ region of the gene. We took the simultaneous presence of Pol II at TSS and H3K4me3 500bp downstream of the TSS to be a reliable indicator of productive transcription. In agreement with this, when assayed by qRT-PCR the abundance of mRNA transcripts randomly selected from the above group varied from 10 to 1% of that for the highly expressed housekeeping gene *RpL32* (*rp49*) and was about two order of magnitude greater than the amount of RNA produced by transcripts randomly s elected from the group that lacked Pol II at the TSS and H3K4me3 around position +500 (Figure S4B). Pol II and H3K4me3 distributions were then similarly mapped in BG3 and D23 cells.

**Supplemental Materials and Methods.**

**Cell culture**

The *Schneider L2* cells (Sg4 clone, also known as SF4) were grown at 25^0^C in 50ml T-flasks to a density of ~5x10^6^ cells/ml in Schneiders *Drosophila* medium (Invitrogen) supplemented with 10% Fetal Bovine Serum (Invitrogen), 100U/ml of Penicillin G, 100μg/ml of Streptomycin sulfate and 292 μg/ml of L-glutamine. The ML-DmBG3-c2 (Ui et al., 1994) and ML-DmD23-c4 cells (Ui et al., 1987) were cultured in the same medium supplemented with 10μg/ml of insulin. The ML-DmBG3-c2 and ML-DmD23-c4 cell lines were obtained from Drosophila Genomics Resource Center (dgrc.cgb.indiana.edu).

**Antibodies**

The source and amount of antibodies used for chromatin immunoprecipitations are detailed in the Table S9. Additional anti-TRX N-ter antibodies were raised in rabbits against a peptide containing amino acids 9-351 of the long isoform fused to GST and affinity purified by passing first through a GST-Sepharose column and then over TRX (9-351)-Sepharose column. Two independent antibodies were used to map each of the following: TRX N-ter, TRX C-ter, and ASH1.

**RNAi**

The RNAi was performed as described by Clemens et al. (2000) with minor modifications. The sequences of PCR primers used to produce DNA template for dsRNA synthesis are indicated in Table S11. ML-DmBG3-c2 cells were subjected to three consecutive treatments with corresponding dsRNA at a ratio of 25μg of dsRNA per 1x10^6^ cells. The cells were grown in regular media for 4 days between each treatment. Four days after the third treatment the cells were harvested and used for preparation of total nuclear protein, total RNA and crosslinked chromatin.

**Western blot analysis**

Total nuclear protein was isolated by first lysing cells in hypotonic buffer containing 10% sucrose, 10mM Tris pH8.0, 10mM NaCl, 3mM MgCl_2_, 2mM DTT and 0.2% Triton X100, followed by 10 min extraction of the nuclear pellet with Sample Buffer (12mM Tris-HCl pH6.8, 5% glycerol, 0.4% SDS, 2.9mM 2-mercaptoethanol, 0.02% bromphenol blue) at 100^0^C. Serial dilutions of protein samples were loaded on 4-18% SDS polyacrylamide gel, separated by electrophoresis, transferred to a PVDF membrane and detected by incubation with primary antibodies at 1:1000 dilution and secondary antibodies conjugated with alkaline phosphatase.

**Primary processing of microarray data**

The TiMAT v2.8.3 open source tiling microarray analysis software package was used for primary data processing. The list of microarray data sets is indicated in the Table S10. To derive the genomic binding profile for a given protein, microarray hybridizations of the DNA from at least two independent ChIP experiments and two matching chromatin inputs were used. Intensities of hybridization were median-scaled to 100. Additional quantile normalization was employed for evaluation of RNAi knock down effects. To compensate for sequence-based variation in hybridization and amplification efficiency, the results were computed as average ChIP to average Input intensity ratios smoothed by taking a trimmed mean over a sliding 675 bp window. Windows containing less then 10 features were excluded from the analysis. Integrated Genome Browser (Affymetrix) was used for visual inspection of the results.

**Definition of bound regions**

For a given protein the genomic set of bound regions was computationally defined as coordinates of clusters of microarray features which satisfied the following two criteria. i) the smoothed ChIP/Input hybridization intensity ratios of the features had to equal or exceed 2, ii) the maximum distance to the nearest neighboring feature with an intensity of at least 2, had to be no less than the selected cutoff. The selection of distance cutoff values varied depending on the kind of protein examined. Shorter cutoff distances were employed in case of proteins like Pol II or H3K4me3 that exhibited localized binding while longer distances were more appropriate for the definition of broadly distributed proteins exemplified by H3K27me3. The distance cutoff values used in current analysis are indicated in Table S12. In addition all regions shorter than 360 bp or for which average ChIP/Input signal within a region was lower than 2 were removed.

The quality of ChIP/chip mapping is critically dependent on the specificity of antibody employed. The primary validation of antibody specificity was conducted as detailed below and in Table S9. For all antibodies against non-histone proteins, with the exception of anti-ASH1 rat mAb which does not work on western-blot, we required the recognition of a nuclear protein of roughly expected size by western-blot in the untreated cells and reduction of the level of this protein after treatment of cells with corresponding dsRNA. We note that for many of the antibodies against non-histone proteins, western-blot analysis revealed a degree of cross-reactivity to proteins whose levels were not affected by specific RNAi treatment. For all anti-histone antibodies (with exception of the monoclonal anti-H3K27me3, which does not work on western-blot) the primary confirmation of specificity was done by comparing results of western-blots with equal amounts of embryonic and recombinant histones. In all cases a single band of histone H3 size was evident on the blot with embryonic but not with recombinant histones. The specificity of anti-H3K27me3 was confirmed by mass-spectrometry of precipitated material.

In addition to primary validation of the antibody specificity, the following approaches were used to ensure the correct mapping of target genes and regions. In the case of PcG target regions or genes only those simultaneously bound by at least one PcG protein and H3K27me3 were considered and only regions bound by at least two PcG proteins and H3K27me3 were used for comparisons between different cell lines. Identification of transcriptionally active genes was discussed above in detail and was principally assured by independent detection of Pol II and H3K4me3 and confirmed by qRT-PCR.

To map ASH1 binding regions, two sets of experiments were done for each cell line, one using a rabbit polyclonal and one using a rat monoclonal antibody. These antibodies were raised against two different protein regions that overlap only by seven amino acids and thus can be considered independent (Sanchez-Elsner et al., 2006). In Sg4 cells, the polyclonal antibody precipitated 242 regions and the monoclonal 69 regions with IP/INPUT ratio equal or greater than 2. About one quarter (23%) of the regions detected with the polyclonal antibody overlapped with those detected by the monoclonal antibody, with a reciprocal overlap ratio of 100%, illustrating the fact that the signals obtained with the monoclonal antibody were generally weaker. A similar relation between the results obtained with the two antibodies was seen for BG3 and D23 cells. The sites common to both antibodies included all the broadest and strongest binding regions (Figure S11). We concluded that these represent true high confidence sites of ASH1 binding and used these for our analysis.

Two different antibodies against TRX C-ter raised by Poux et al. (2002) and Beisel at al. (2007) were used to map the C-terminal moiety of TRX. Both antibodies consistently detected TRX C-ter binding at PREs (Figure S12A). However, outside of PcG target regions the results obtained with the two antibodies showed clear differences in specificity (Figure S12B). For example in Sg4 cells the antibodies from Beisel at al. (2007) detected 758 sites outside PcG target regions that bind TRX with IP/Input ratio equal or greater than 2. At the same enrichment cutoff 90% of these regions were also detected with antibodies from Poux et al. (2002). However the antibodies from Poux et al. (2002) detected many more TRX binding sites, 83% of which were not detected by the Beisel at al. (2007) antibodies. This discrepancy was not due to the difference in the relative strength of the two antibodies as the two detected sites with comparable spectra of enrichment. The overwhelming majority of the TRX binding sites unique for the Poux et al. (2002) antibodies corresponded to the TTS of transcriptionally active genes (80% of all active promoters precipitate with this antibody). Importantly the intensities of ChIP signals at PREs but not at active TSS were reduced upon TRX RNAi. Overall, we concluded that the Poux et al. (2002) antibodies react with some additional protein bound at active promoters. We considered only the regions simultaneously detected by the two antibodies as true sites of TRX C-ter binding.

To verify the accuracy of mapping of the TRX N-ter we compared the results of mapping in BG3 cells done with two independently raised antibodies, one described by Kuzin et al. (1994) and another described above. The results were essentially the same and throughout the work the two antibodies were used interchangeably. Thus the antibodies produced by Kuzin et al., (1994) were used for primary mapping of TRX N-ter distribution in the three cell lines. The antibodies produced in this work were used to compare the distributions of TRX N-ter in BG3 cells before and after TRX or ASH1 RNAi.

**Computational definition of presumptive PREs**

The subset of presumptive PREs in Sg4 cells was defined as 1 kb regions centered around a binding peak of E(Z) which resided within 250bp of a peak of PSC and overlapped by PC bound region. The E(Z) and PSC peaks were defined as follows. As a first step, smoothed ChIP/Input signals for each protein were filtered by excluding the ones with ChIP/Input ratio below selected cutoff (2-fold enrichment for E(Z) and 2.2-fold enrichment for PSC). We have chosen the slightly more conservative cutoff ratio for PSC to compensate for the weak nonspecific activity against an unrelated chromosomal protein present in our anti-PSC antibody preparation. The filtered sets of signals were further transformed into “binding intervals”, by grouping together the signals separated by a gap of 2000 bp or less. As a second step the “binding peaks” within the “binding intervals” were determined by i) further smoothing the signals via local polynomial regression. For small intervals, additional positions to the left and the right of the interval were used to permit smoothing. ii) Identifying local maxima in the resulting signal. To be called a peak, such a maximum had to exhibit a drop-off on both the left and right which exceeded 0.1 for log_2_(ChIP/Input). iii) “Binding intervals” for which the local maximum of the smoothed signal fell beyond the interval boundaries were assigned a peak corresponding to the maximum of the smoothed signal within the interval. The comparison of E(Z) and PSC peaks between each other and with PC bound regions resulted in 171 regions, which were then visually inspected in the genome browser. A region of Chromosome 2L, positions 20170586-20171586, was revealed to be an artifact of the poor representation of this genomic interval on our tiling arrays. This region was excluded and the resulting set of 170 computational PREs was used for further analysis.

**Evaluation of statistical significance of the overlap between two sets of regions**

To estimate the probability that the extent of overlap between two sets of bound regions was observed by chance (p-value) the following algorithm was used. The genomic positions of regions within both sets of intervals were randomized and the extent of overlapping between the two sets computed. The procedure was repeated 1000 times and the number of randomizations that resulted in the extent of overlapping equal to or greater than that observed for the original sets of intervals was counted (n). From this the p-value was defined as:

p=n*10^-3^

in cases when n=0 the p-value was considered to be less than 0.001. Only statistically significant overlaps with p-values < 0.001 were considered in this study.

**Supplemental References**

1. Barski, A., Cuddapah, S., Cui, K., Roh, T.Y., Schones, D.E., Wang, Z., Wei, G., Chepelev, I., Zhao, K. (2007). High-resolution profiling of histone methylations in the human genome. Cell *129*, 823-837.
2. Beisel, C., Buness, A., Roustan-Espinosa, I. M., Koch, B., Schmitt, S., Haas, S. A., Hild, M., Katsuyama, T., and Paro, R. (2007). Comparing active and repressed expression states of genes controlled by the Polycomb/Trithorax group proteins. Proceedings of the National Academy of Sciences *104*, 16615-16620.
3. Clemens, J.C., Worby, C.A., Simonson-Leff, N., Muda, M., Maehama, T., Hemmings, B.A., and Dixon, J.E. (2000) Use of double-stranded RNA interference in *Drosophila* cell lines to dissect signal transduction pathways. Proc. Natl. Acad. Sci. USA *97*, 6499-6503.
4. Dellino, G. I., Schwartz, Y. B., Farkas, G., McCabe, D., Elgin, S. C. R., and Pirrotta, V. (2004). Polycomb silencing blocks transcription initiation. Mol Cell *13*, 887-893.
5. Kahn, T. G., Schwartz, Y. B., Dellino, G. I., and Pirrotta, V. (2006). Polycomb complexes and the propagation of the methylation mark at the Drosophila Ubx gene. J Biol Chem *281*, 29064-29075.
6. Kuzin, B., Tillib, S., Sedkov, Y., Mizrokhi, L., and Mazo, A. (1994). The Drosophila trithorax gene encodes a chromosomal protein and directly regulates the region-specific homeotic gene fork head. Genes Dev *8*, 2478-2490.
7. Pokholok, D.K., Harbison, C.T., Levine, S., Cole, M., Hannett, N.M., Lee, T.I., Bell, G.W., Walker, K., Rolfe, P.A., Herbolsheimer, E., et al. (2005). Genome-wide map of nucleosome acetylation and methylation in yeast. Cell *122*, 517-527.
8. Poux, S., McCabe, D., and Pirrotta, V. (2001).Recruitment of components of Polycomb Group chromatin complexes in *Drosophila*. Development *128*, 75-85.
9. Poux, S., Horard, B., Sigrist, C. J. A., and Pirrotta, V. (2002). The Drosophila Trithorax protein is a coactivator required to prevent re-establishment of Polycomb silencing. Development *129*, 2843-2893.
10. Sanchez-Elsner, T., Gou, D., Kremmer, E., and Sauer, F. (2006). Noncoding RNAs of Trithorax Response Elements Recruit Drosophila Ash1 to Ultrabithorax. Science *311*, 1118-1123.
11. Schwartz, Y. B., Kahn, T. G., Nix, D. A., Li, X.-Y., Bourgon, R., Biggin, M., and Pirrotta, V. (2006). Genome-wide analysis of Polycomb targets in Drosophila melanogaster. Nat Genet *38*, 700-705.
12. Ui, K., Ueda, R., Miyake, T. (1987). Cell lines from imaginal discs of Drosophila melanogaster. In Vitro Cell. Dev. Biol. *23*, 707-711.
13. Ui, K., Nishihara, S., Sakuma, M., Togashi, S., Ueda, R., Miyata, Y., Miyake, T. (1994). Newly established cell lines from Drosophila larval CNS express neural specific characteristics. In Vitro Cell. Dev. Biol. A. *30*, 209-216.
